# Supplementary material for: Vibrational wavepacket dynamics in Fe carbene photosensitizer determined with femtosecond X-ray emission and scattering
Source: Nat Commun. 2020 Jan 31;11:634. doi: 10.1038/s41467-020-14468-w (PMC6994595; doi:10.1038/s41467-020-14468-w)
Supplement: Supplementary file 1 — Supplementary Information [file 41467_2020_14468_MOESM1_ESM.pdf]

Supplementary Information

Vibrational wavepacket dynamics in Fe carbene photosensitizer determined with femtosecond X-ray emission and scattering

Kunnus et al.

Supplementary Note 1: Kα and Kβ XES spectra of $[Fe(bmip)_2]^{2+}$ and the model complexes

Kα and Kβ XES spectra from various model complexes were measured at the SSRL in order to evaluate the effect of spin state and ligand environment to the spectra. Each series of experiments was carried out during a single beamtime to allow direct comparison of the spectra (in particular the energy shift). Ligand environment dependence of Kα/Kβ XES spectra is shown in Supplementary Figure 1. Low spin $[Fe(bmip)_2]^{2+}$ is compared to four other low spin Fe complexes in a series of $[Fe(bpy)_N(CN)_{6-2N}]^{2N-4}$ (N=0 – 3) with decreasing ligand field strength. Fe spin state dependence of Kα/Kβ XES spectra is shown in Supplementary Figure 2. Kβ XES FePc spectrum is from Ref. [1].

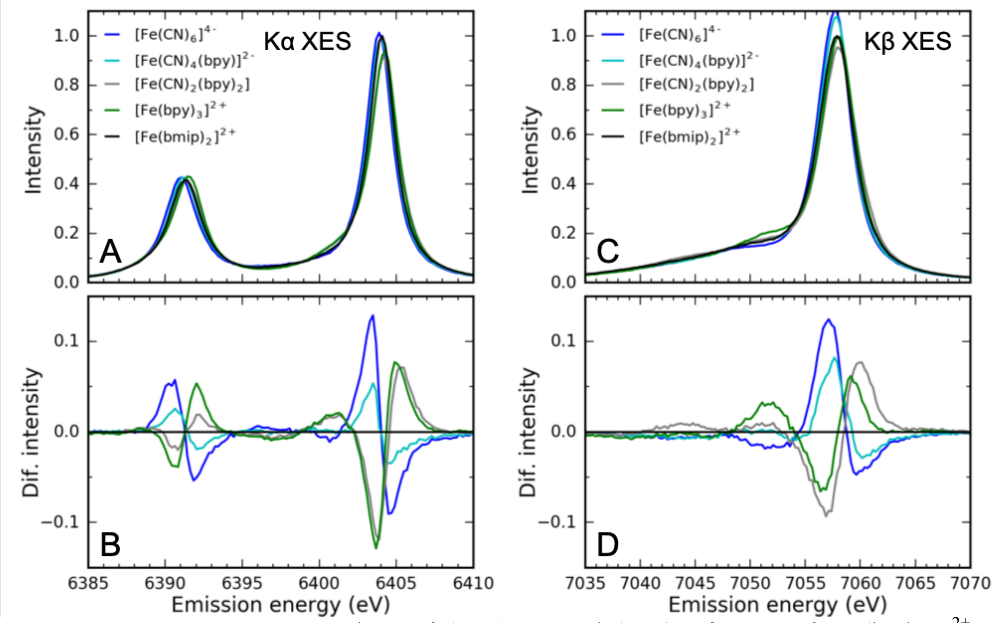

Supplementary Figure 1. Comparison of (A,B) Kα and (C,D) Kβ XES of $[Fe(bmip)_2]^{2+}$ with $[Fe^{II}(bpy)_N(CN)_{6-2N}]^{2N-4}$ (N=0 – 3). Spectra are normalized to the same area.

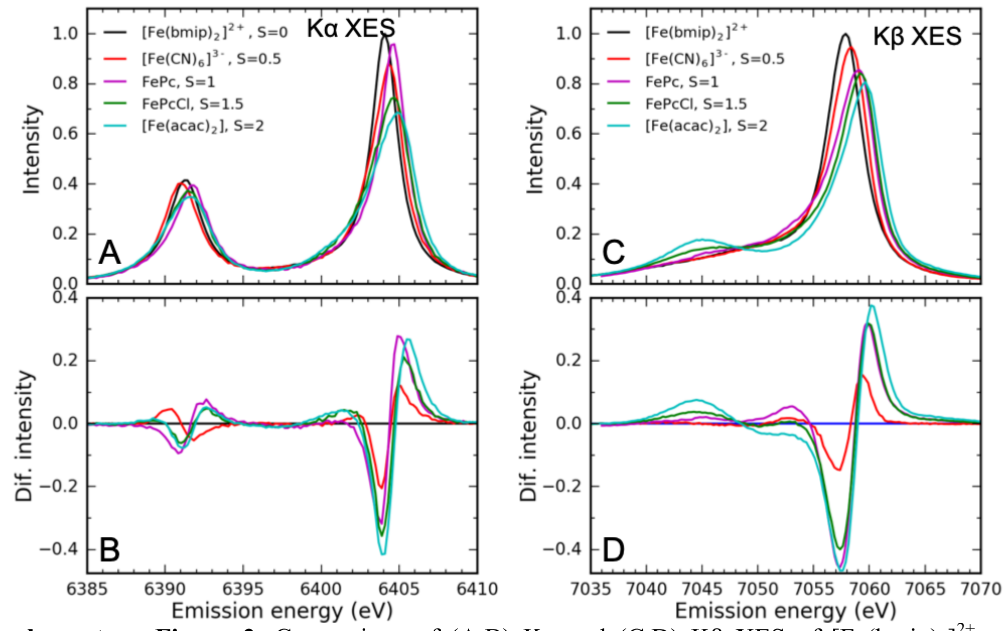

Supplementary Figure 2. Comparison of (A,B) Kα and (C,D) Kβ XES of $[Fe(bmip)_2]^{2+}$ with complexes with different Fe spin state S. Spectra are normalized to the same area.

Supplementary Note 2: Correction of Kβ XES spectra measured at the LCLS

Misalignment of one of the four analyzer crystals of the multicrystal spectrometer during the LCLS experiment resulted in distorted Kβ XES spectra. In order to remove this distortion, we express the measured (uncorrected) spectrum S_{uncorr} as a sum of two correct spectra S_{corr} that are shifted with respect to each other:

$$S_{uncorr}(x) = (1-r)S_{corr}(x) + rS_{corr}(x-s)$$

Here, r is the relative intensity of the misaligned (shifted) spectrum and s is the shift. Following from the properties of the δ-function, we can express S_{uncorr} as a convolution

$$S_{uncorr}(x) = S_{corr}(x) * D(x)$$

where S_{corr} is convolved with a function D describing the misalignment:

$$D(x) = (1-r)\delta(x) + r\delta(x-s)$$

Misalignment correction is therefore equivalent to a deconvolution problem and can be readily solved by applying the convolution theorem. Correct spectrum can be thus calculated from a measured (uncorrected) spectrum with a following formula

$$S_{corr}(x) = FT^{-1}\left\{\frac{FT\{S_{uncorr}(x)\}}{FT\{D(x)\}}\right\}$$

Direct application of the convolution theorem to carry out the deconvolution is possible here because D is a sum of δ-functions and is noise free. Therefore, singularities do not appear in the division with $FT\{D\}$. In general, deconvolution of noisy data requires regularization (e.g. by imposing some smoothness criterium to the result), this is fortunately not necessary here (due to the specific form of D). The correction procedure includes finding appropriate relative intensity r and shift s of the two delta peaks in D. This is achieved by comparison to the known $[Fe(bmip)_2]^{2+}$ ground state spectrum measured at the SSRL with an identical spectrometer. In Supplementary Figure 3 we demonstrate the application of this procedure to the ground state Kβ XES spectrum. We found that the misaligned portion of the spectrum is shifted by 20 pixels and it corresponds to 28% of the total intensity. Exactly the same D as shown in Supplementary Figure 3 was then applied to correct all the time-resolved XES spectra at different time delays. This is valid because the correction procedure defined above is a linear operation and does not depend on the shape of S_{uncorr}. Therefore, it can be applied to spectra with any shape (i.e. not only the ground state spectrum, but also to all the transient spectra). Also, it is evident from the power spectrum of D, $FT\{D\}^{*}FT\{D\}$ in Supplementary Figure 3 that this procedure does not involve any filtering (e.g. low pass) or smoothing of the data. We demonstrate latter by comparing the residuals of S_{corr} taken with respect to the reference spectrum and the residuals of S_{uncorr} taken with respect to a "distorted" reference spectrum (Supplementary Figure 4).

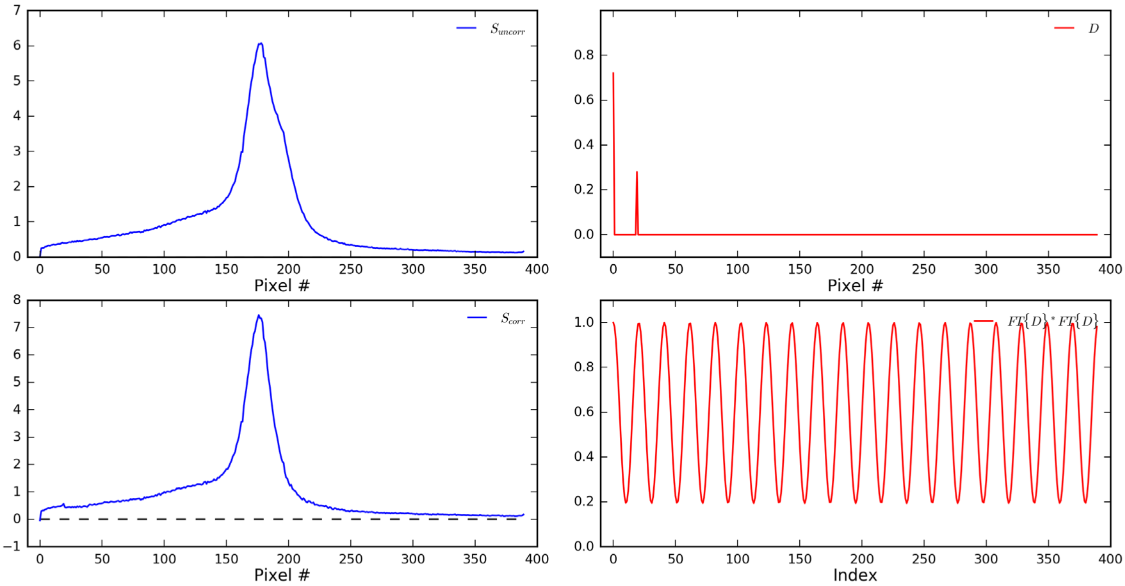

Supplementary Figure 3. Correction of spectral distortion due to misalignment of one of the spectrometer crystals. Two delta peaks in D are shifted by 20 pixels and their relative heights are 0.72/0.28.

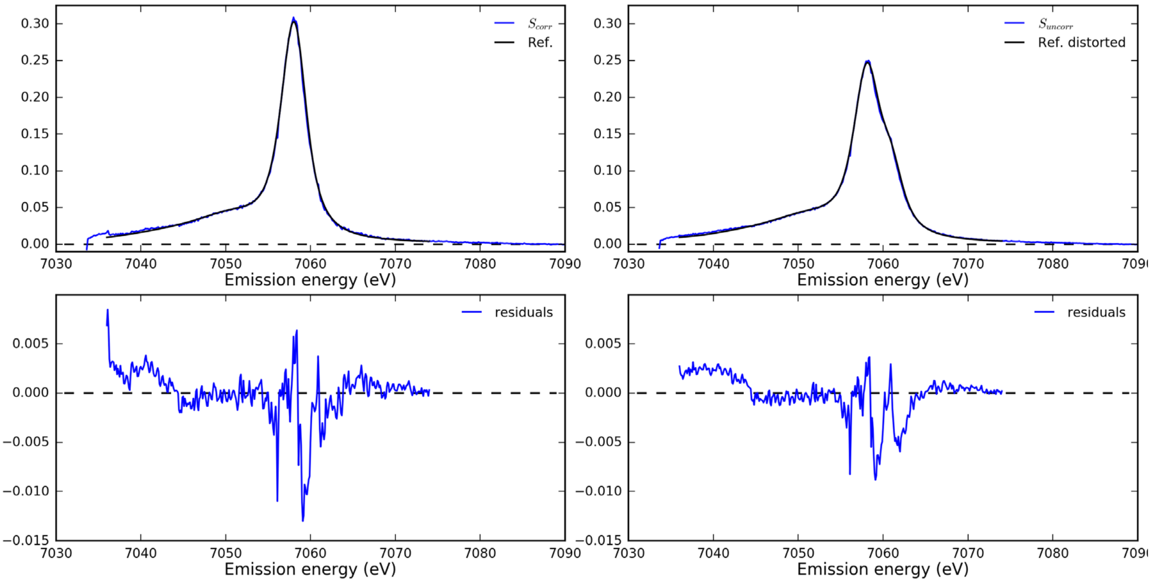

Supplementary Figure 4. Comparison of noise characteristics of corrected and uncorrected XES spectra. Reference spectrum (black line) corresponds to the $[Fe(bmip)_2]^{2+}$ spectrum measured at the SSRL.

Supplementary Note 3: Kβ XES singular value decomposition

Singular value decomposition (SVD) of the $[Fe(bmip)_2]^{2+}$ time-resolved Kβ XES data is displayed in Supplementary Figure 5. It is evident that the first SVD component includes all the time-dependent signal, whereas higher SVD components only include the noise. Therefore, there is no changes in the shape of the time-resolved Kβ XES differences signal. Comparison of the data and the reconstruction based on the first SVD component is shown Supplementary Figure 6.

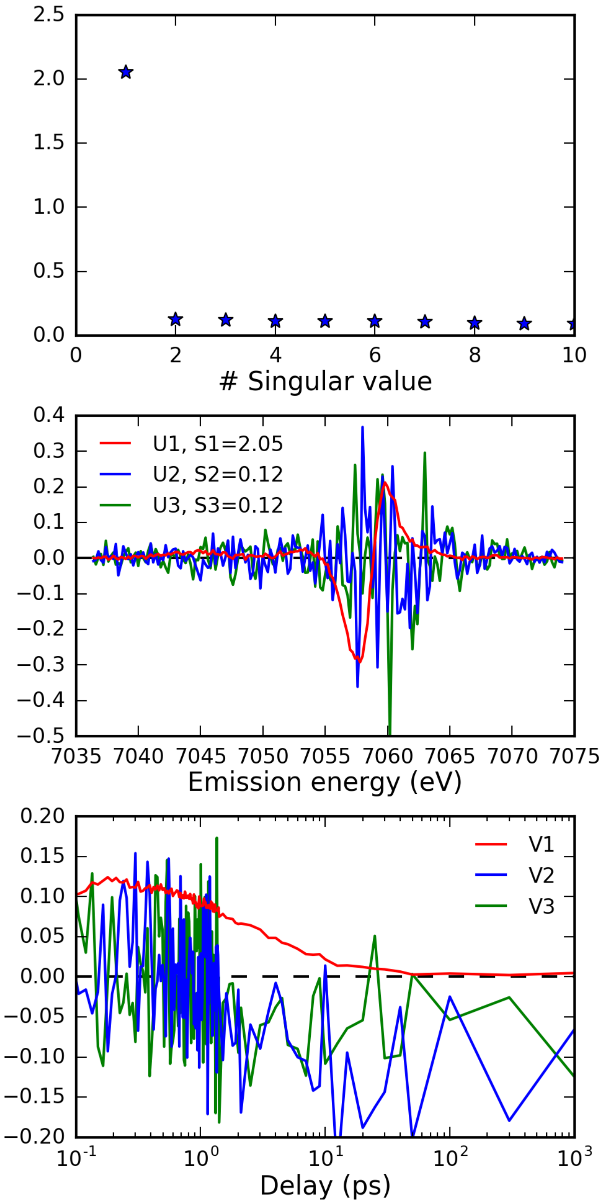

Supplementary Figure 5. Singular value decomposition (SVD) of the $[Fe(bmip)_2]^{2+}$ time-resolved Kβ XES data. (Top) Singular values and (middle) the first three respective column vectors U and (bottom) row vectors V.

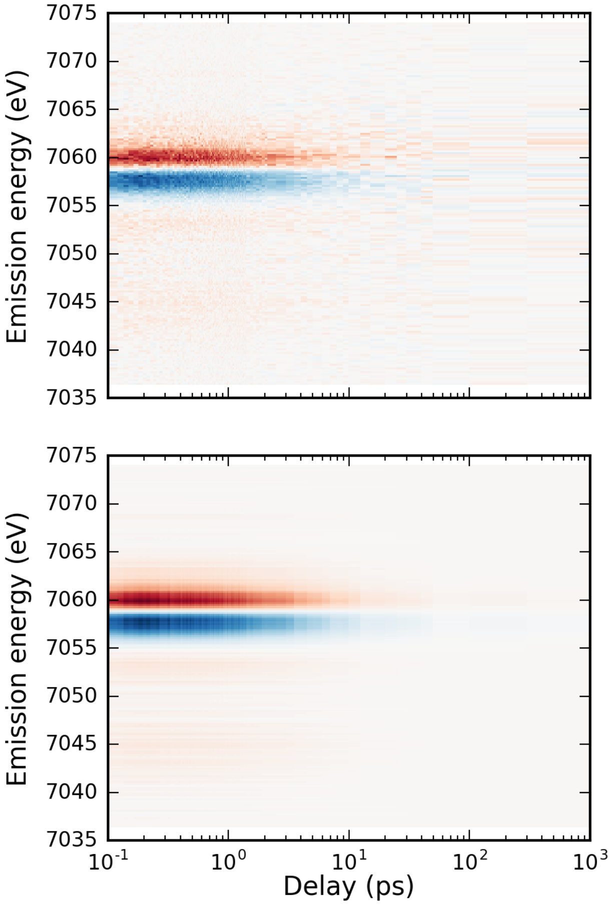

Supplementary Figure 6. Comparison of the $[Fe(bmip)_2]^{2+}$ time-resolved Kβ XES data (top) and the reconstruction based on the first SVD component (bottom).

Supplementary Note 4: Kβ XES oscillatory dynamics

We fitted independently the h delay traces in order to quantitatively evaluate the Kβ XES oscillatory signal. In this analysis the time constants of the Kβ delay trace were not constrained by the Kα data and the MLCT* and the ^{3}MLCT intensities were allowed to be different. This allows most optimal description of the non-oscillatory Kβ signal and extraction of the oscillatory component. Results of the fits are shown in Supplementary Figure 7. Fit of the Kβ oscillatory signal yields a period of 280±12 fs and a damping constant of 266±110 fs. We find that within the experimental uncertainties, the relative amplitude (~1% signal modulation of the total XES signal) and the period of the oscillations in Kα and Kβ XES are the same. However, there is a discrepancy between the damping constants. We believe that this could be explained by the changing noise level in the Kβ delay trace that seems to be higher after 0.5 ps. Note that overall, the Kβ XES noise level is $\sigma = 0.4\%$, which is nearly an order-of-magnitude higher than the $\sigma = 0.05\%$ in Kα XES experiment.

Although the higher Kβ XES noise level does not allow drawing definite conclusions, we propose that the origin of the Kβ oscillatory signal is the same as for the Kα oscillatory signal. That is, linear shifting of the Kβ XES emission energies due to a small relative displacement of the 1s and 3p core-ionized states PESs.

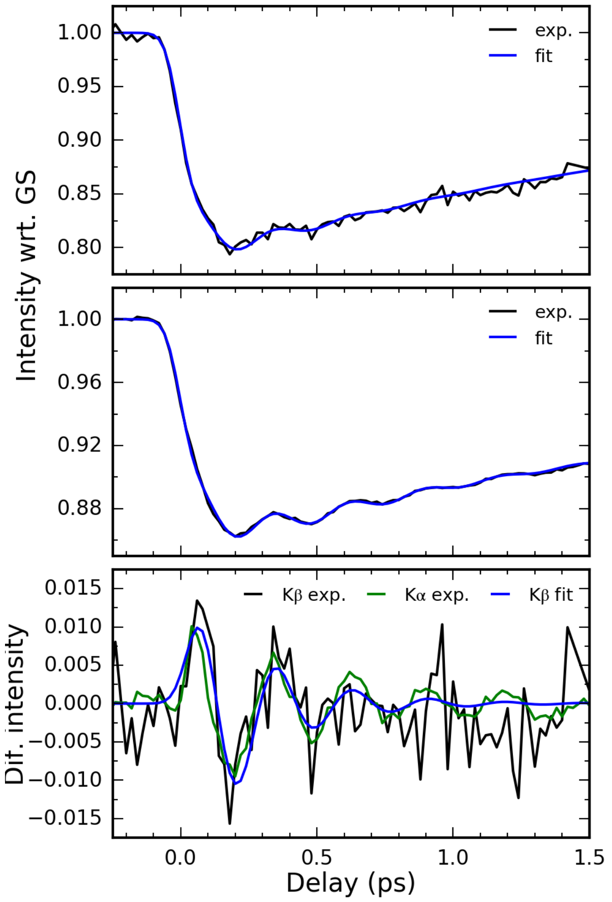

Supplementary Figure 7. Independent analysis of the time-resolved Kβ and Kα XES data. (Top) Fits of Kβ and (middle) Kα early time dynamics exhibiting oscillatory signal. (F) Oscillatory Kβ XES signal after subtraction of the non-oscillatory part and comparison with the Kα XES oscillatory signal.

Supplementary Note 5: Fluence dependence of UV-visible transient absorption

UV-visible transient absorption (TA) of $[Fe(bmip)_2]^{2+}$ in acetonitrile measured at three different 400 nm laser fluences are displayed in Supplementary Figure 8. The data was measured with the same experimental set-up as in Ref. [2]. We find no significant fluence dependent changes in the observed dynamics. Differences in the shape of TA spectra at 0-0.1 ps is likely due to a presence of the cross-phase modulation (CPM) signal. Difference intensities at 0.5-2 ps show linear dependence with the laser fluence (Supplementary Figure 9). A thorough analysis of the TA data is presented in the next section.

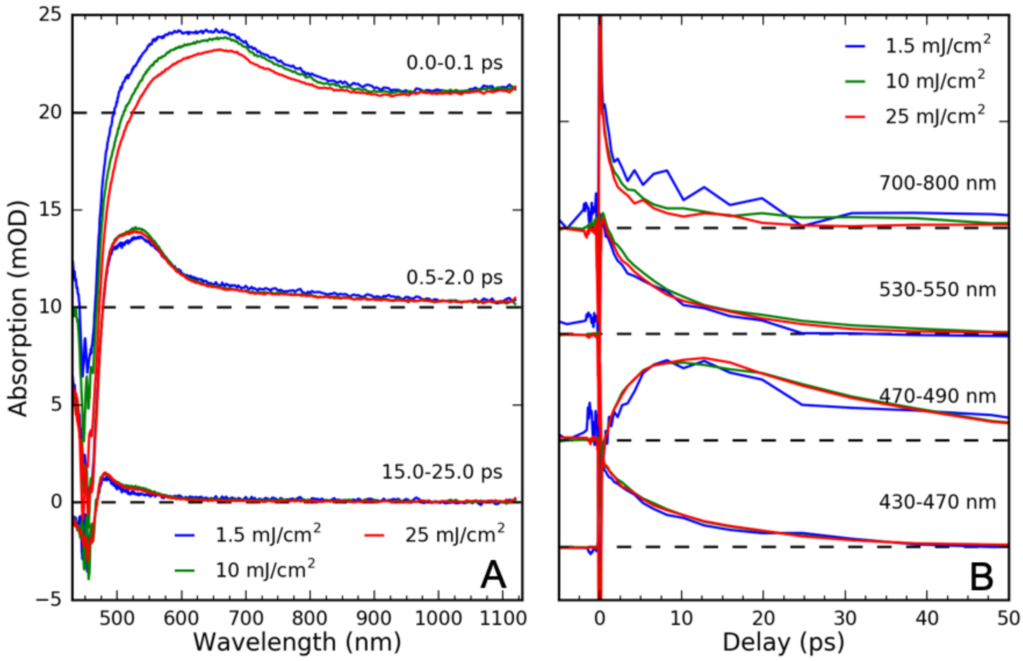

Supplementary Figure 8. Pump fluence dependence of the UV-visible transient absorption (TA) data. (A) Difference TA spectra at selected delay regions. The spectra are normalized with the laser fluence for comparison. (B) Delay traces at selected wavelength region. The traces are normalized with the laser fluence and scaled with the following factors (bottom to top): -1, 5, 2.5, 10.

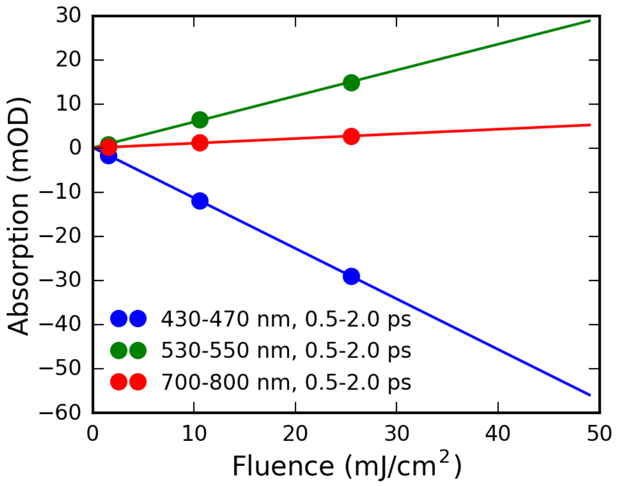

Supplementary Figure 9. Pump fluence dependence of the UV-visible transient absorption signal in different wavelength regions at 0.5-2.0 ps.

Supplementary Note 6: Global analysis of UV-visible transient absorption

UV-visible transient absorption (TA) of $[Fe(bmip)_2]^{2+}$ in acetonitrile has been previously measured with 485 nm excitation [2]. A single 9 ps excited state time constant was assigned there to ^{3}MLCT decay, whereas 11 ps time constant was observed for the ground state bleach recovery. Below we present the TA data of $[Fe(bmip)_2]^{2+}$ in acetonitrile that is excited with 400 nm light to enable direct comparison with the XES and XSS data (Supplementary Figure 10-S12). This data has been measured with three different laser fluences: 1.5 mJ/cm^2, 10 mJ/cm^2 and 25 mJ/cm^2 (0.5 kHz, 200 μm pump diameter; same experimental set-up as in Ref. [2]). Close inspection of the 2D TA data reveals that the dynamics is not a single exponential. Fitting of delay traces at different wavelength regions with a biexponential parallel kinetic model revels time constant from 1 to 20 ps. In order to consistently describe these dynamics over the whole detected wavelength range we carried out a global analysis of the TA data. The excited state lifetimes were fixed to the values retrieved from the XES. The ^{3}MC and ^{3}MLCT lifetimes are thus 1.5 ps and 9 ps, respectively. Additionally, we included a component corresponding to a hot ground state. Results of this global analysis for the three different laser fluences are shown in Supplementary Figure 10-S12. We find that this model successfully describes the data at all three laser fluences. In Supplementary Figure 13 we display a comparison of a hot ground state difference spectrum extracted from the fit and a difference spectrum of a simulated hot ground state spectrum. Good agreement between these confirms the assignment of the ~20 ps signal to the hot ground state species. We therefore conclude that XES and XSS data measured with 45 mJ/cm^2 laser fluence at the LCLS is consistent with the lower fluence TA experiments. In addition to the excited state dynamics, we find that TA is also sensitive to the temperature of the recovered ground state $[Fe(bmip)_2]^{2+}$ molecules.

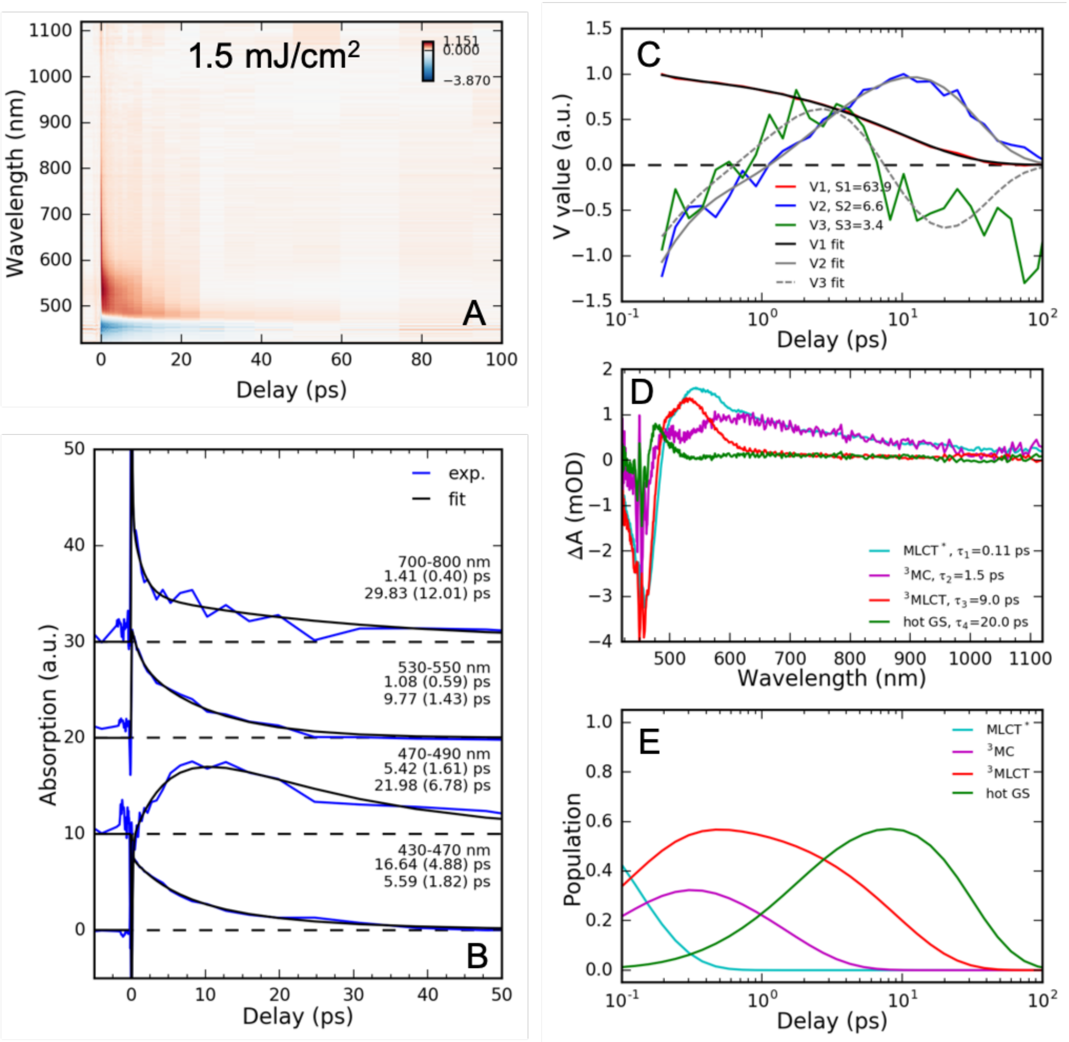

Supplementary Figure 10. Analysis of the UV-visible transient absorption (TA) with 1.5 mJ/cm^2 fluence. (A) Delay time dependence of the difference absorption spectra. (B) Delay traces in four selected wavelength regions and the fits with unconstrained biexponential parallel kinetics model. Cross phase modulation at 0 ps is excluded from the fit. Delay traces are scaled (bottom to top): -1, 5, 2.5, 10. (C-E) Global analysis of the TA data using the same kinetic model as for XES/XSS with an addition hot ground state population (C) Fits of the first three SVD row vectors V. (D) Species associated spectra assigned to the three excited states based on the XES analysis and to the hot ground state (GS). (E) Population dynamics corresponding to the excited states and the hot ground state.

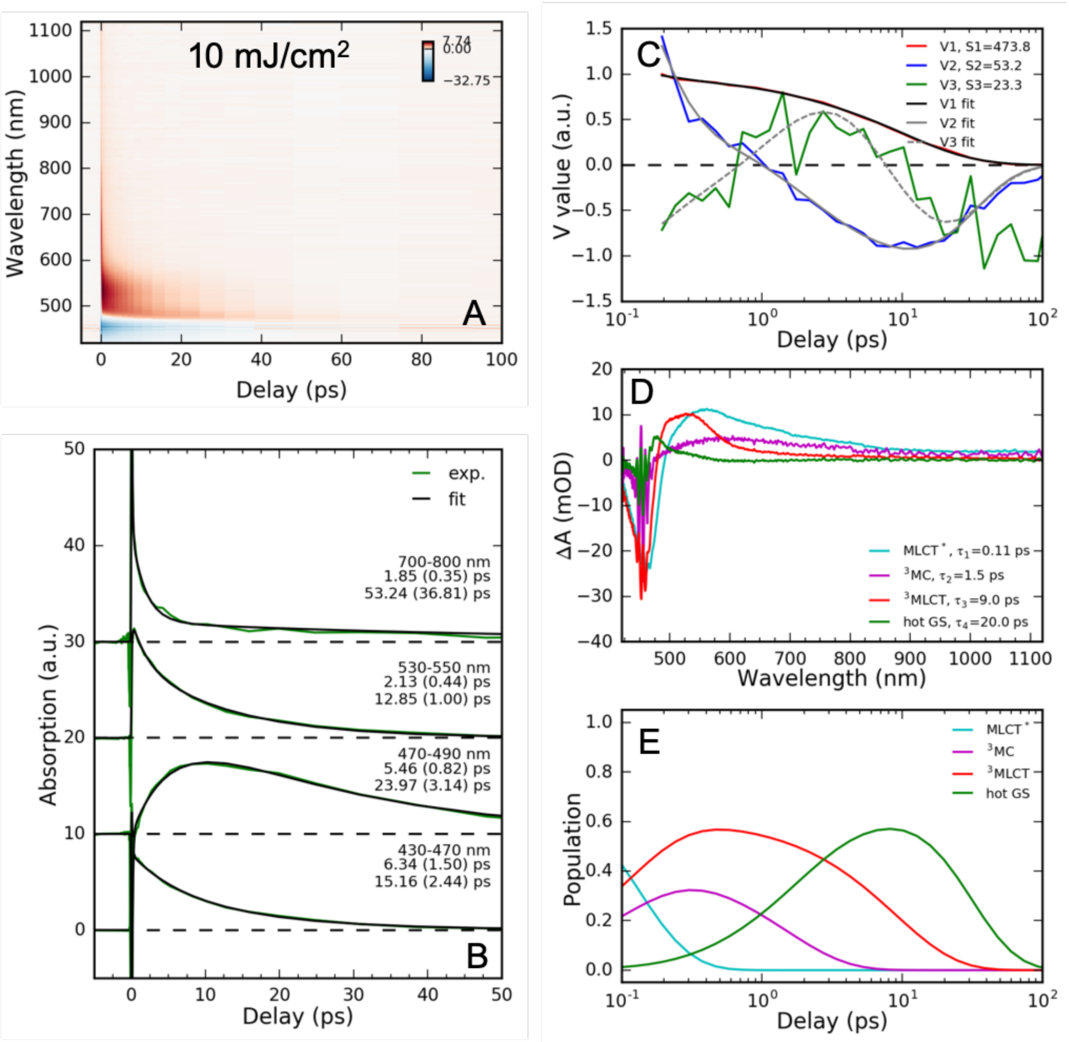

Supplementary Figure 11. Analysis of the UV-visible transient absorption (TA) with 10 mJ/cm^2 fluence. (A) Delay time dependence of the difference absorption spectra. (B) Delay traces in four selected wavelength regions and the fits with unconstrained biexponential parallel kinetics model. Cross phase modulation at 0 ps is excluded from the fit. Delay traces are scaled (bottom to top): -1, 5, 2.5, 10. (C-E) Global analysis of the TA data using the same kinetic model as for XES/XSS with an addition hot ground state population (C) Fits of the first three SVD row vectors V. (D) Species associated spectra assigned to the three excited states based on the XES analysis and to the hot ground state (GS). (E) Population dynamics corresponding to the excited states and the hot ground state.

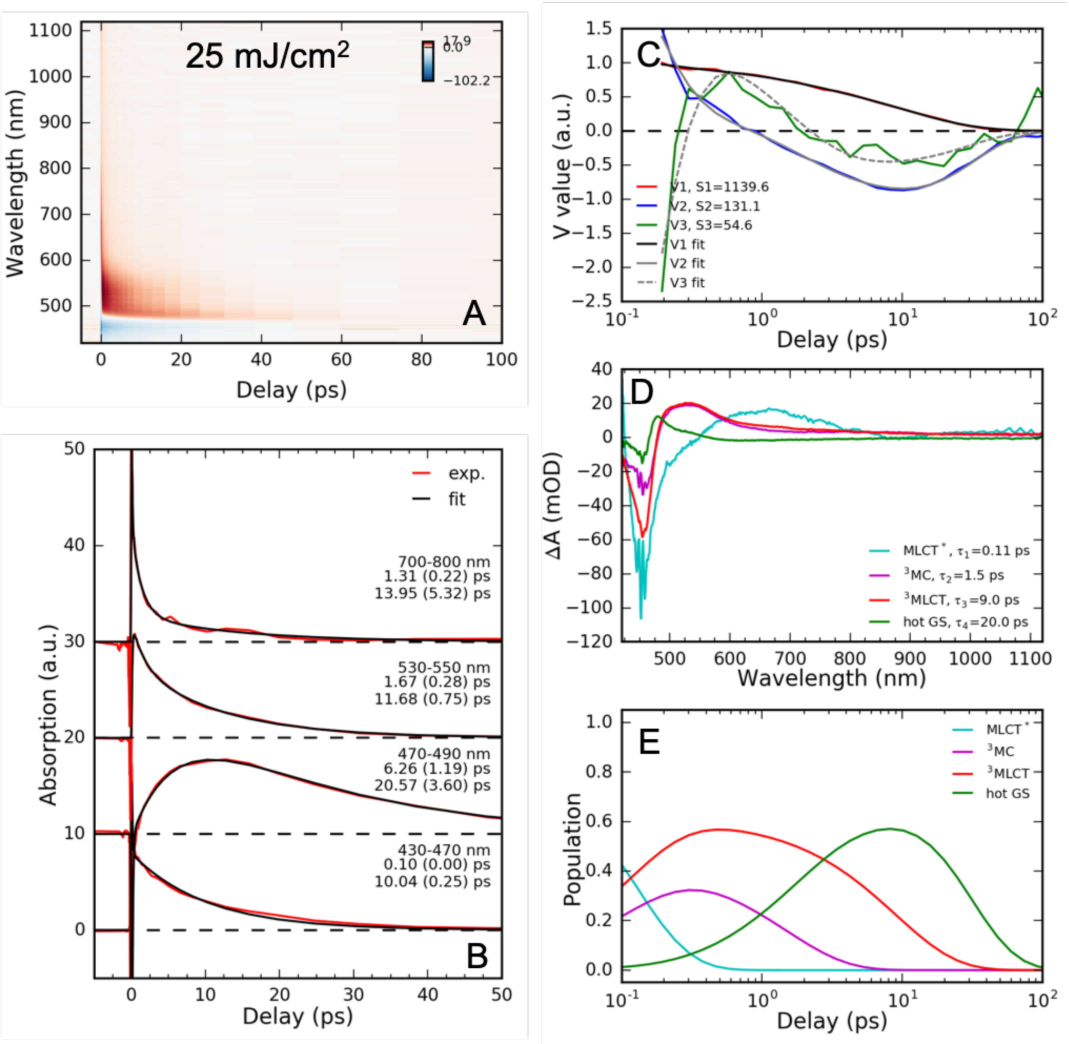

Supplementary Figure 12. Analysis of the UV-visible transient absorption (TA) with 25 mJ/cm^2 fluence. (A) Delay time dependence of the difference absorption spectra. (B) Delay traces in four selected wavelength regions and the fits with unconstrained biexponential parallel kinetics model. Cross phase modulation at 0 ps is excluded from the fit. Delay traces are scaled (bottom to top): -1, 5, 2.5, 10. (C-E) Global analysis of the TA data using the same kinetic model as for XES/XSS with an addition hot ground state population (C) Fits of the first three SVD row vectors V. (D) Species associated spectra assigned to the three excited states based on the XES analysis and to the hot

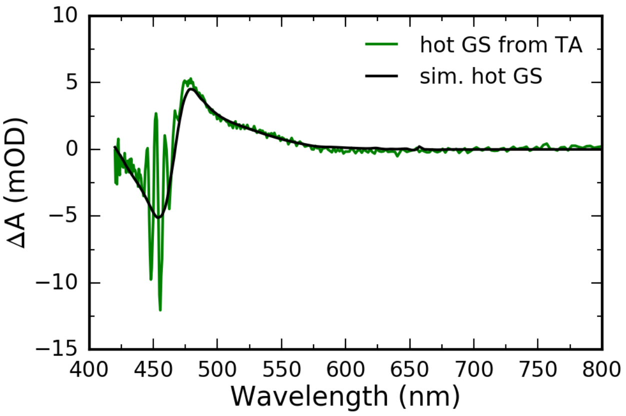

Supplementary Figure 13. Comparison of a hot ground state spectrum extracted from the global analysis of the 10 mJ/cm^2 UV-visible transient absorption (TA) data and a difference spectrum of a simulated hot ground state spectrum (scaled). The simulated spectrum is a sum of two spectra (two temperatures) that are red-shifted by 13 nm and broadened with 10 nm and 100 nm FWHM Gaussian, respectively.

Supplementary Note 7: UV-visible transient absorption oscillatory dynamics

The oscillatory dynamics in $[Fe(bmip)_2]^{2+}$ was first reported by Liu *et al.* [2] in UV-visible transient absorption with 485 nm pump (Supplementary Information of Ref. [2]). In Supplementary Figure 14 is a comparison of the UV-visible transient absorption (TA) and the Kα XES kinetics. TA experiment was carried out at 400 nm pump and 520 nm probe wavelengths (same experimental set-up as in Ref. [2]). Both the TA and the Kα XES show oscillatory dynamics. Comparison and fitting of these dynamics is shown in Supplementary Figure 15. Fit of the TA oscillatory signal yields a period of 280±3 fs and a damping constant of 1480±580 fs. The period is same in both TA and X-ray experiment and can be therefore assigned to the same underlying dynamics. Note that the TA fit shows deviations from the data in delay region from -0.3 ps to 0.4 ps (resulting in overestimation of the fitted damping constant). This is most likely due to a cross phase modulation that is often observed in TA experiments.

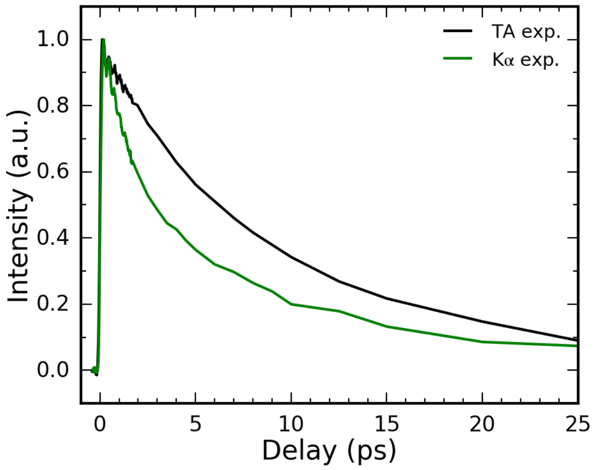

Supplementary Figure 14. Comparison of the UV-visible transient absorption (TA) and Kα XES delay traces. In both experiments the pump wavelength was 400 nm. The TA delay trace was detected at 520 nm and the Kα XES delay trace was detected at 6404.3 eV.

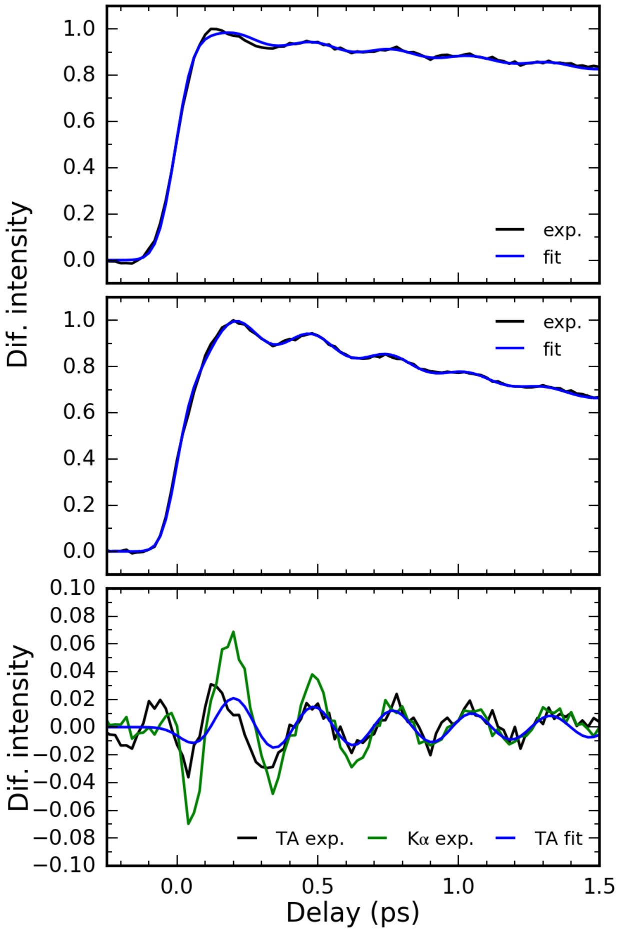

Supplementary Figure 15. Independent analysis of the time-resolved TA and Kα XES delay traces. (Top) Fits of TA and (middle) Kα early time dynamics exhibiting oscillatory signal. (F) Oscillatory TA signal after subtraction of the non-oscillatory part and comparison with the Kα XES oscillatory signal.

Supplementary Note 8: Excitation yield determination

In order to determine the photoexcitation yield of $[Fe(bmip)_2]^{2+}$ solute molecules during the time-resolved experiment at the LCLS we measured also time-resolved Kβ XES of a reference sample. Latter measurement was carried out immediately after the $[Fe(bmip)_2]^{2+}$ experiment to guarantee consistent laser excitation conditions. Reference sample was $[Fe(btbip)_2]^{2+}$ [btbip = 2,6-bis(3-*tert*-butyl-imidazole-1-ylidine)-pyridine]. This sample was recently investigated with XSS [3]. After a MLCT excitation this complex relaxes ultrafast to a long-lived ^{5}MC state and this allows it to use for a robust excitation yield determination. Global analysis of the $[Fe(btbip)_2]^{2+}$ difference Kβ XES kinetics is shown in Supplementary Figure 16. Comparison of the data and the fit are in Supplementary Figure 17. We find 0.2 ps MLCT lifetime, 279±33 ps ^{5}MC lifetime and a complete GS recovery. The ^{5}MC lifetime agrees with the 260 ps lifetime reported in Ref. [3]. In Supplementary Figure 18 the excitation yield of $[Fe(btbip)_2]^{2+}$ is determined to be 71%, estimated from the amplitude of the difference Kβ XES spectrum and the Kβ' intensity at 7045 eV in particular.

Below we relate the measured excitation yield of $[Fe(btbip)_2]^{2+}$ to the excitation yield of $[Fe(bmip)_2]^{2+}$. Firstly, we take into account the different concentrations and linear absorption cross sections of the respective solutions:

$[Fe(btbip)_2]^{2+}$: OD(50 μm, 15.8 mM, 400 nm) = 0.29

$[Fe(bmip)_2]^{2+}$: OD(50 μm, 20.3 mM, 400 nm) = 0.59

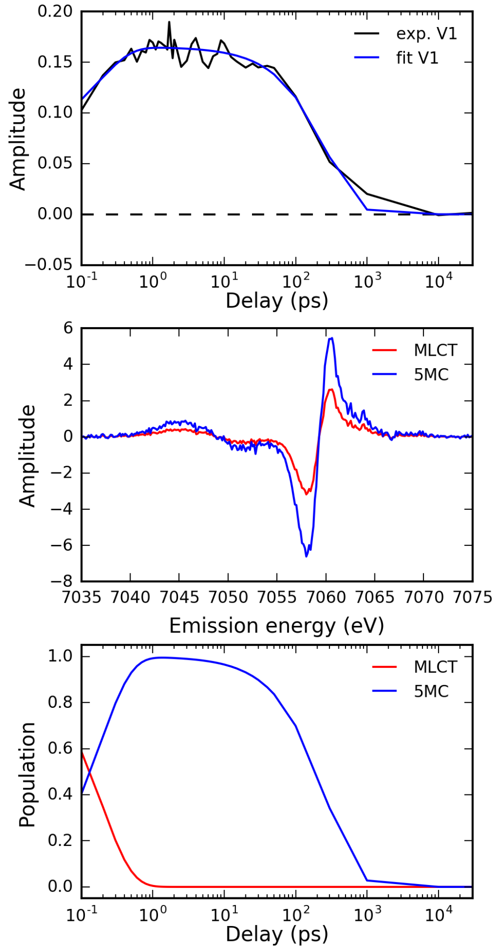

Supplementary Figure 16. Global analysis of the $[Fe(btbip)_2]^{2+}$ Kβ XES. (Top) Two component sequential fit of the row vector V1 corresponding to the first Kβ XES SVD component. (Middle) Species associated spectra of the two kinetic components, assigned to the MLCT and the ^{5}MC states. (Bottom) Relative populations of the MLCT and the ^{5}MC. The ^{5}MC lifetime of $[Fe(btbip)_2]^{2+}$ is 279±33 ps.

Secondly, because we cannot assume a linear excitation regime, we take into account the lowest order non-linear absorption effects. These are: 1) ground state depletion, 2) stimulated emission (SE), and 3) excited state absorption (ESA). SE cross section of ^{1}MLCT state at 400 nm is equal to the ground state absorption at 400 nm. However, SE is significantly suppressed due to ultrafast vibrational relaxation and ^{1}MLCT->^{3}MLCT intersystem crossing during the duration of the pump laser pulse (45 fs FWHM). Latter has been measured to be faster than 20 fs in $[Fe(bpy)_3]^{2+}$ [4]. We therefore introduce 10 fs time constant that depopulates ^{1}MLCT and populates ^{3}MLCT. We assume that the ^{3}MLCT cannot be stimulated to the ground state with 400 nm light. ESA cross section we estimate from the short time-scale UV-visible transient absorption spectra in Supplementary Figure 19. By assuming that the ESA cross section is a slowly varying function the wavelength, we estimate that the ESA cross section is very likely <50% of the ground state cross section. In Supplementary Figure 20 we have simulated the excitation yield dependence from the laser fluence for different ESA cross sections (assumed to be same for ^{1}MLCT and ^{3}MLCT). We find that ESA cross section influences the total excitation yield only a small amount and the dominant non-linear effect is ground state depletion that results in a saturated absorption profile.
Laser fluence during both $[Fe(btbip)_2]^{2+}$ and $[Fe(bmip)_2]^{2+}$ experiments was 45 mJ/cm^2. Based on the information in Supplementary Figure 20 we can now relate the experimentally determined 71% excitation yield of $[Fe(btbip)_2]^{2+}$ to the excitation yield of $[Fe(bmip)_2]^{2+}$. By taking ESA cross section equal to 50% of the ground state cross section, we find that the $[Fe(bmip)_2]^{2+}$ excitation yield is $0.71 \times (0.7/0.6) = 0.83$. Note that is very similar to the excitation yield if one would assume linear absorption regime: $0.71 \times (1.1/0.93) = 0.84$. Given the number of assumptions and experimental uncertainties in this estimation, we conclude that the excitation yield of $[Fe(bmip)_2]^{2+}$ is very likely 84±10%. Therefore, the XES population kinetics analysis was carried out with excitation yields in a range from 74% to 94%. As presented in the main manuscript, variation of the excitation yield within this range does not change the conclusion we draw.

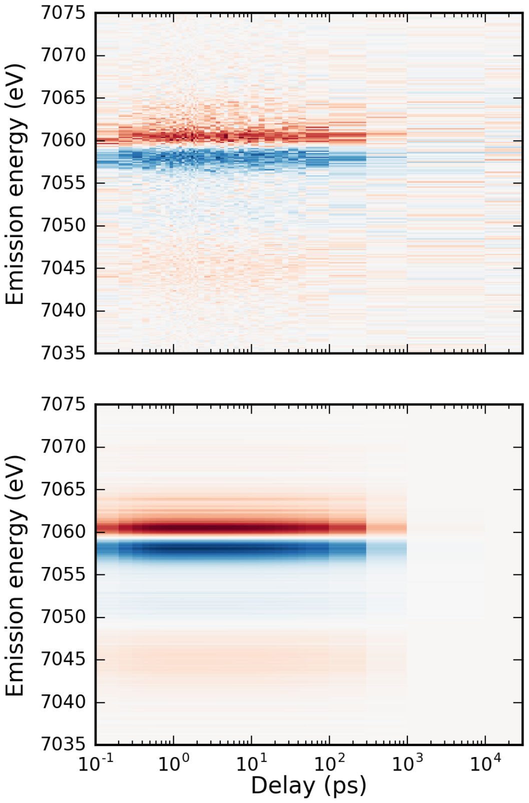

Supplementary Figure 17. Comparison of the $[Fe(btbip)_2]^{2+}$ time-resolved Kβ XES data (top) and the global fit (bottom)

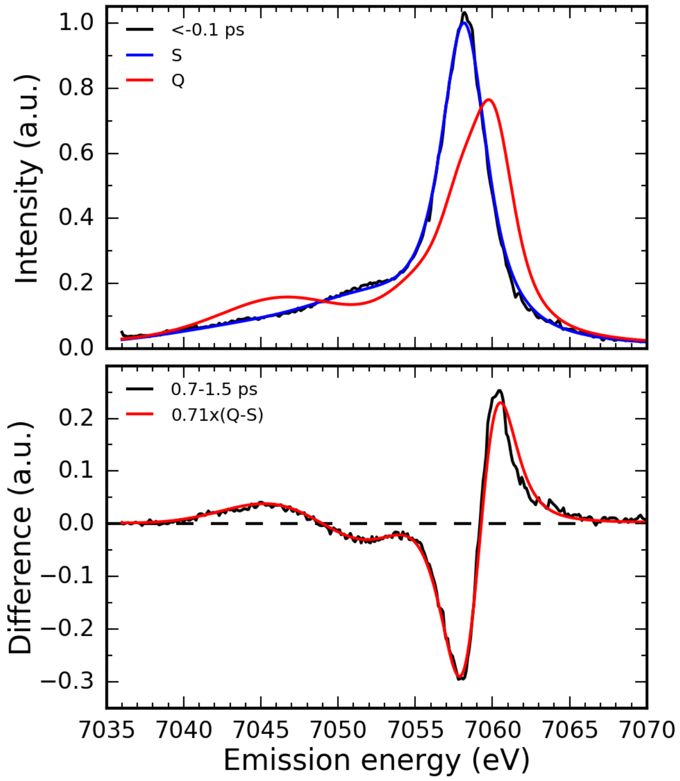

Supplementary Figure 18. Excitation yield determination from $[Fe(btbip)_2]^{2+}$ Kβ XES. (Top) Singlet ground state of $[Fe(btbip)_2]^{2+}$ before photoexcitation (black line), singlet model spectrum (blue line), quintet ^{5}MC model spectrum (red line). (Bottom) Difference spectrum from 0.7 – 1.5 ps (black line) and the scaled difference spectrum of singlet and quintet states (red line).

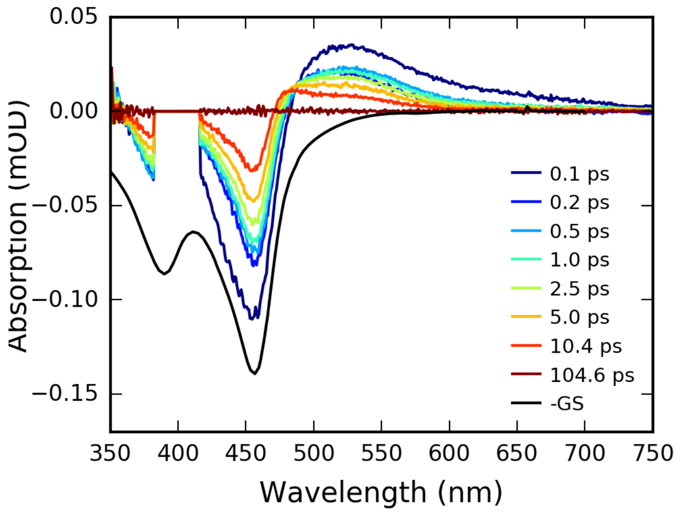

Supplementary Figure 19. Comparison of UV-visible transient absorption (TA) data and a ground state (GS) spectrum of $[Fe(bmip)_2]^{2+}$ in acetonitrile.

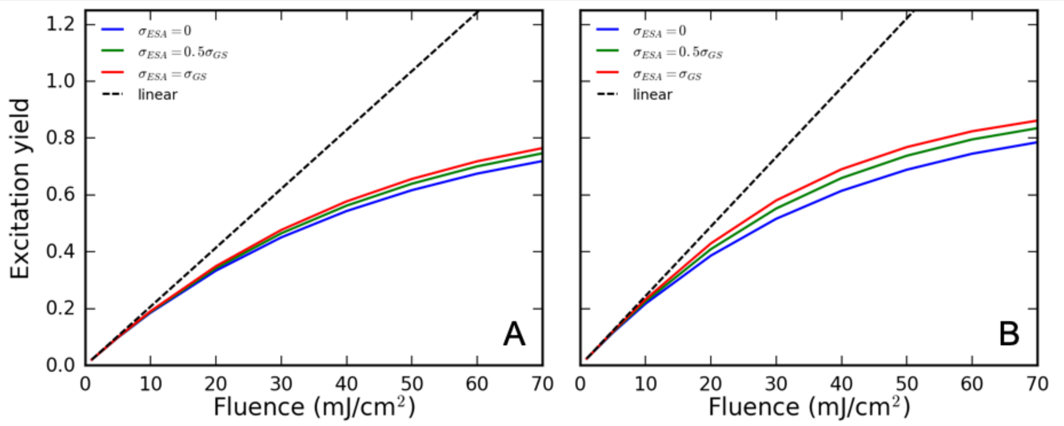

Supplementary Figure 20. Excitation yield simulations in high laser fluence conditions. (A) $[Fe(btbip)_2]^{2+}$ solution in MeCN, OD(50µm,15.8mM,400nm) = 0.29. (B) $[Fe(bmip)_2]^{2+}$ solution in MeCN, OD(50µm,20.3mM,400nm) = 0.59. Laser pulse duration is 45 fs. Simulations consider excitation into a ^{1}MLCT state that interconverts to ^{3}MLCT with 10 fs. Simulated emission cross section of ^{3}MLCT is zero. Excited state absorption cross sections of ^{1}MLCT and ^{3}MLCT are assumed to be equal.

Supplementary Note 9: Comparison of different kinetic models

Different kinetic models were tested to fit the Kα and Kβ XES data. Below we present the results from four kinetic models that were test. The Kα and Kβ XES delay traces were fitted simultaneously and in all the kinetic models the excitation yield was fixed to 84%. All the models succeed in capturing time-dependent dynamics, except the model displayed in Supplementary Figure 21. There the ground state recovery is modeled by a single time constant. It is therefore clear that at least two excited states are required to model the observed decay. A three-state sequential model in Supplementary Figure 22 captures the time-dependent intensity changes. However, it requires the long-lived ^{3}MLCT state to have too small difference amplitudes, also significantly smaller from the short-lived MLCT* state. Alternative to a sequential model is presented in Supplementary Figure 23. This model is the same as discussed in the main manuscript and it includes branching of the MLCT* into ^{3}MC and ^{3}MLCT that subsequently decay parallel to the ground state. Possible variation of this kinetics is shown in Supplementary Figure 24. There we have tested the likely scenario that the ^{3}MLCT does not decay to the ground state directly, but via the ^{3}MC. Because the ^{3}MLCT lifetime is significantly longer than the ^{3}MC lifetime, then no considerable ^{3}MC population builds-up at late delays. We find that both models in Supplementary Figure 23 and Fig, S24 are consistent with the observed data.

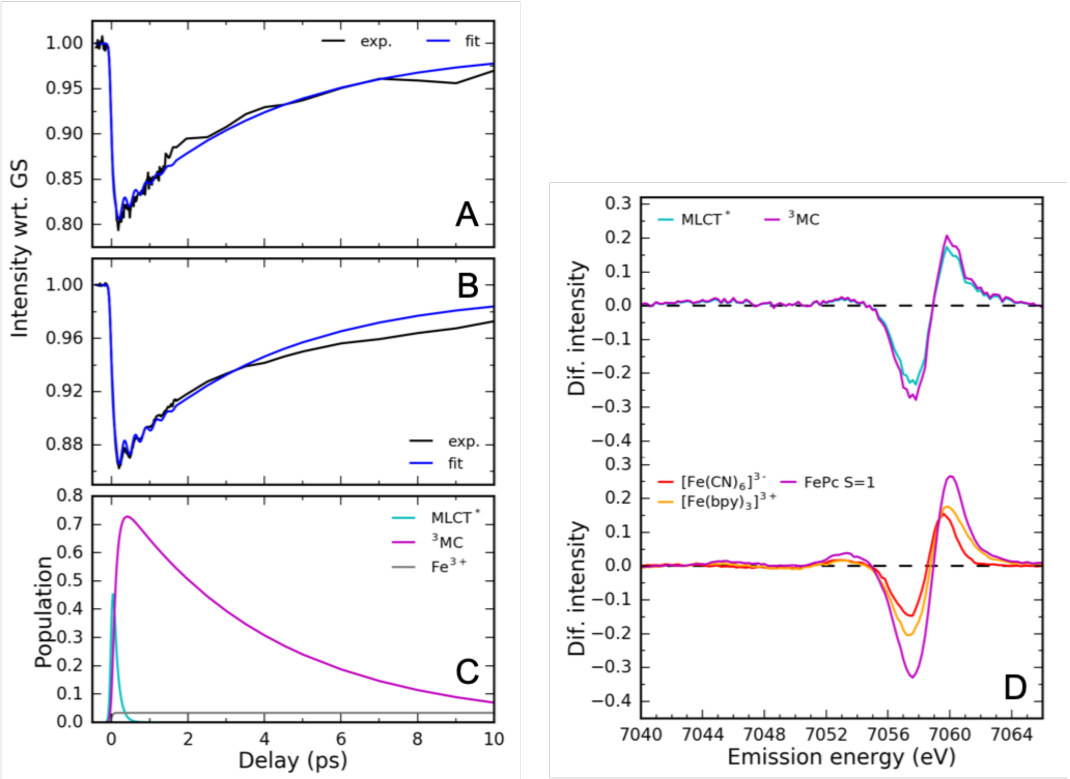

Supplementary Figure 21. Simultaneous fitting of the time-resolved Kβ and Kα XES data with a two-state sequential kinetic model. (A) Fit of the Kβ delay trace between 7056 – 7058.5 eV. (B) Fit of the Kα delay trace at 6404.3 eV. (C) Population of the excited state. (D) Comparison of the excited state difference Kβ spectra with the difference spectra of the model complexes. Note that the fit does not describe the experimental dynamics accurately. τ(MLCT*) = 0.11 ps, τ(^{3}MC) = 4.0 ps.

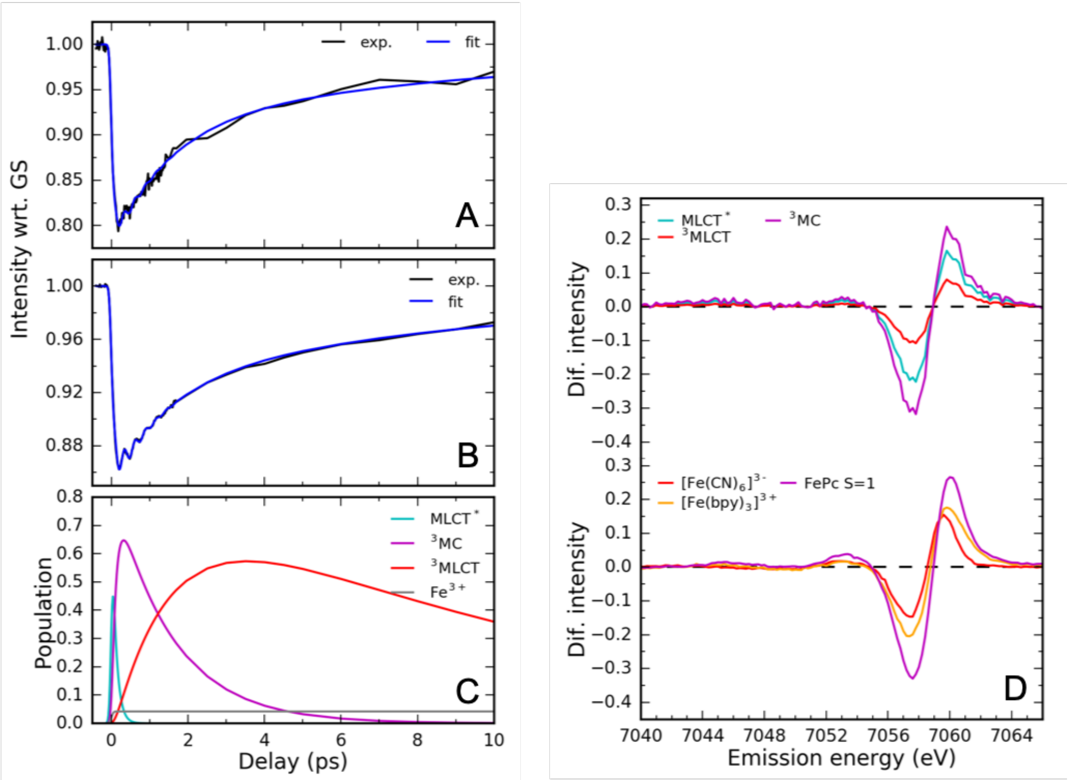

Supplementary Figure 22. Simultaneous fitting of the time-resolved Kβ and Kα XES data with a three-state sequential kinetic model. (A) Fit of the Kβ delay trace between 7056 – 7058.5 eV. (B) Fit of the Kα delay trace at 6404.3 eV. (C) Population of the excited state. (D) Comparison of the excited state and the model complexes difference Kβ spectra. τ(MLCT*) = 0.11 ps, τ(^{3}MC) = 1.5 ps, τ(^{3}MLCT) = 10.4 ps.

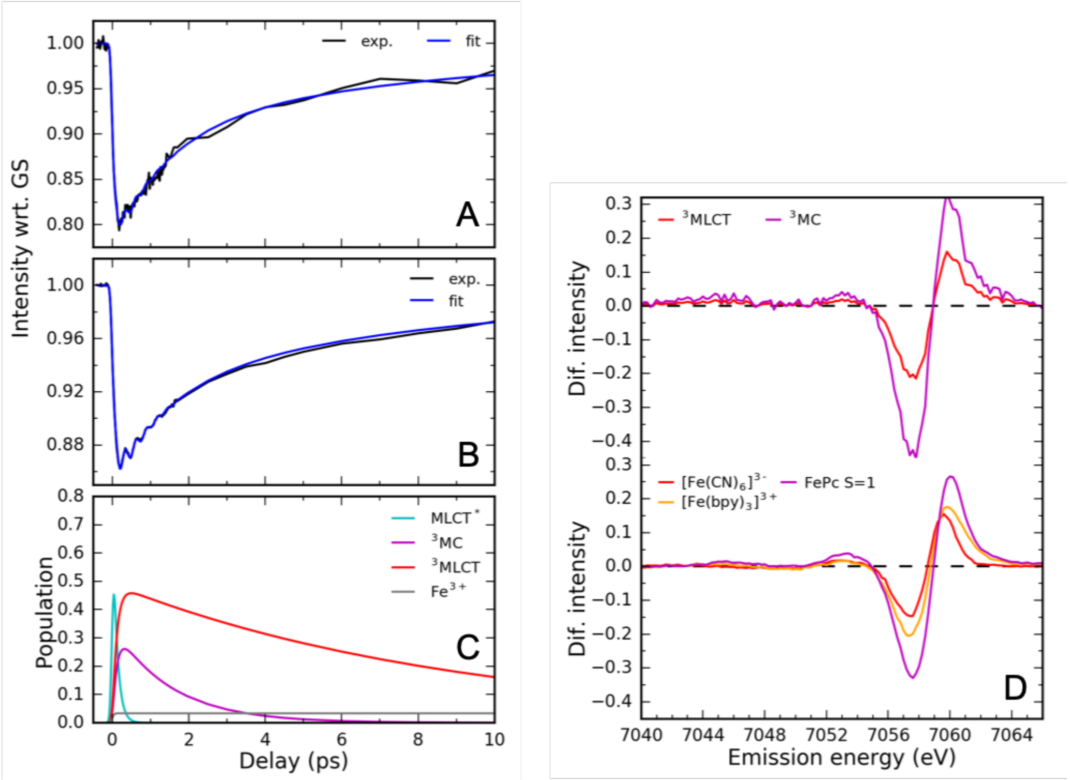

Supplementary Figure 23. Simultaneous fitting of the time-resolved Kβ and Kα XES data with a branching and parallel decay kinetic model. (A) Fit of the Kβ delay trace between 7056 – 7058.5 eV. (B) Fit of the Kα delay trace at 6404.3 eV. (C) Population of the excited state. (D) Comparison of the excited state and the model complexes difference Kβ spectra. τ(MLCT*) = 0.11 ps, τ(^{3}MC) = 1.5 ps, τ(^{3}MLCT) = 9 ps, r(^{3}MC) = 40%.

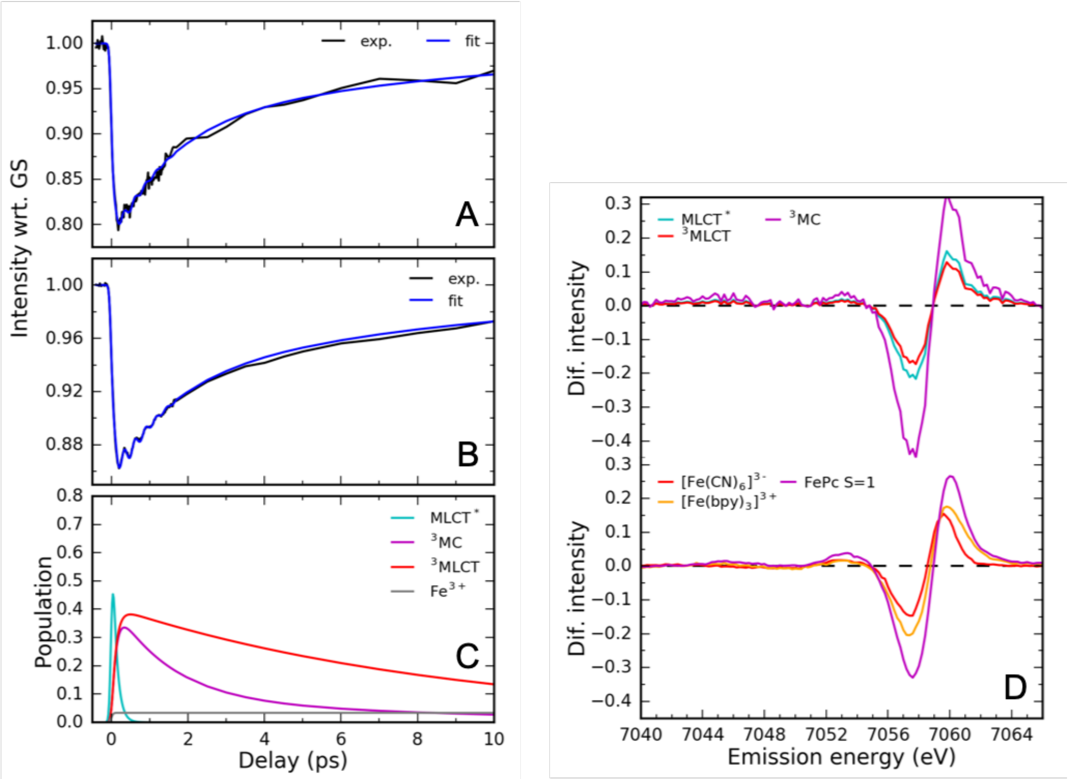

Supplementary Figure 24. Simultaneous fitting of the time-resolved Kβ and Kα XES data with a modified branching and parallel decay kinetic model. Decay of ^{3}MLCT to the ground state is via ^{3}MC. (A) Fit of the Kβ delay trace between 7056 – 7058.5 eV. (B) Fit of the Kα delay trace at 6404.3 eV. (C) Population of the excited state. (D) Comparison of the excited state and the model complexes difference Kβ spectra. τ(MLCT*) = 0.11 ps, τ(^{3}MC) = 1.5 ps, τ(^{3}MLCT) = 9 ps, r(^{3}MC) = 50%.

Supplementary Note 10: Anisotropic XSS signal

Time-resolved XSS difference signal was decomposed into isotropic and anisotropic components following the procedure described in Ref. [5]. The anisotropic XSS signal is shown in Supplementary Figure 25. We found that the anisotropic XSS can be fully described by the optical Kerr-effect (OKE) signal induced in the acetonitrile. The time-dependence of the OKE signal is in an agreement with the dynamics observed in Ref. [6]. The fast initial dynamics <200 fs is slightly non-exponential, however the data can be described with a very good accuracy by two parallel exponential decays with 110 fs and 1.4 ps time constants (in agreement with Ref. [6]). Fit with this kinetics is shown in Fig, S25C. Decay time constants were kept fixed in the fit and we used the OKE signal to check the time-zero (t_0) and the width of the Gaussian instrument response function (IRF). The best fit values are t_0 = 20±10 fs and FWHM=110±20 fs.

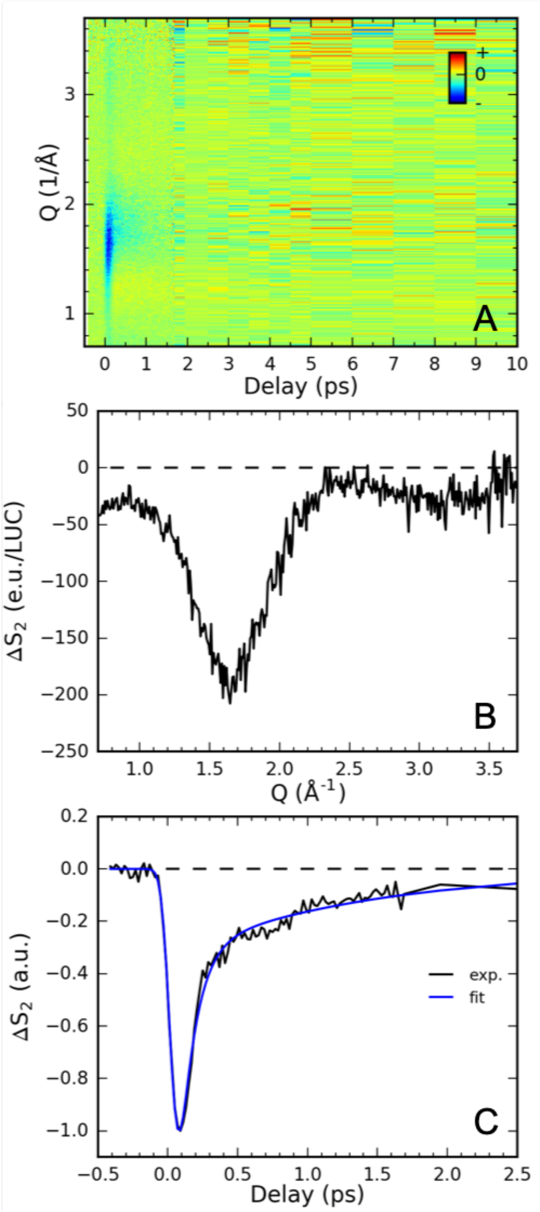

Supplementary Figure 25. Anisotropic XSS signal corresponding to the optical Kerr-effect (OKE) induced in acetonitrile. (A) Overview of time-resolved anisotropic signal from -0.5 ps to 10 ps. (B) Average anisotropic signal between 0 to 0.2 ps. (C) Time-dependent signal amplitude between 1.5 to 1.9 Å^{-1} and a fit with a parallel biexponential decay (τ_1=110 fs, τ_2=1.4 ps; see text).

Supplementary Note 11: Global analysis of XSS

Isotropic XSS data is shown in Supplementary Figure 26. Q-axis was calibrated using a reference acetonitrile heat signal (Supplementary Figure 27A) [7]. Comparison in Supplementary Figure 27A also shows that at time scales >50 ps only solvent heat signal remains. The time-dependence of the solvent heat amplitude was analyzed by fitting the solvent heat signal at each time delay (Supplementary Figure 27B). We found that solvent heating can be well described with two exponential rise times corresponding to 0.35 ps and 13.2 ps. These phenomenological time constants are a convolution of multiple time scales related to solute internal conversion (MLCT*->^{3}MLCT->GS and MLCT*->^{3}MC->GS) and vibrational energy distribution (IVR) processes, as well as fast solvation (<1 ps) and slower vibrational cooling time scales of the various solute electronic state populations (~10 ps). In addition, direct excitation of solvent via OKE process contributes to ultrafast solvent heating (Supplementary Figure 25). The biexponential fit in Supplementary Figure 27B is directly used in global fitting of the whole isotropic XSS data to model the time-dependence of the solvent heat signal (see below).

Singular value decomposition (SVD) was used to separate XSS data into components with different time-dependence (Supplementary Figure 28). The first three SVD components are above the noise level and were therefore selected for the analysis (Supplementary Figure 29). Global analysis was performed by simultaneously fitting the relative amplitude of each time-dependent component for each SVD row vector V. The six time-dependent components were

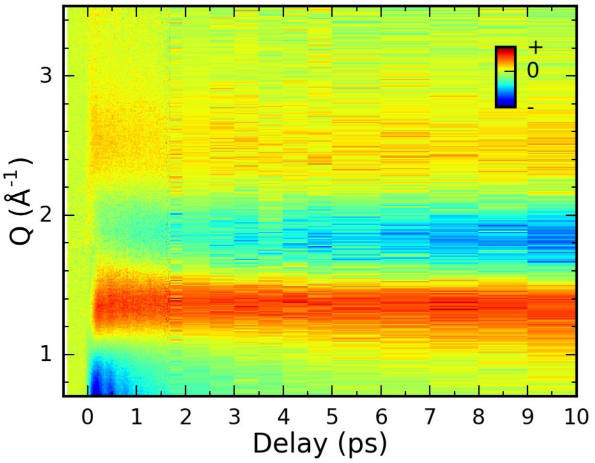

Supplementary Figure 26. Isotropic time-resolved XSS signal from -0.5 ps to 10 ps.

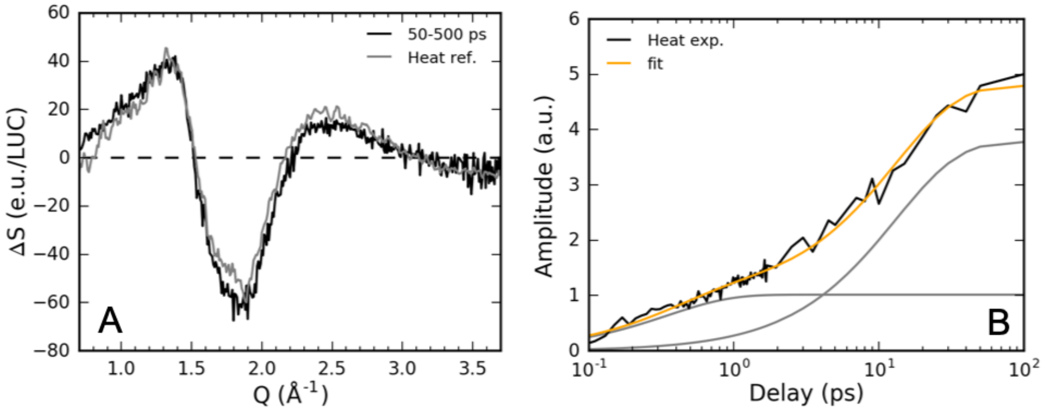

Supplementary Figure 27. XSS solvent heat signal. (A) Average XSS difference signal between 50-500 ps (black) compared with a reference acetonitrile heat signal. (gray). (B) Time-dependence of the solvent heat amplitude extracted by fitting the signal between 50-500 ps at each time delay (black) and a fit with two exponential rise times (orange). Gray lines show the two exponential components with 0.35 ps and 13.2 ps time constants with a relative amplitude ratio of 1 to 3.7.

MLCT*, ^{3}MLCT and ^{3}MC populations (from Kα/Kβ XES analysis), oscillatory component (from Kα XES fit), solvent heating and a delay gaussian function that describes the residual ultrafast dynamics that is not captured by the other components. Presence of this residual ultrafast non-exponential component is a result highly non-equilibrium (ballistic) dynamics at early time scales (<200 fs), possibly related to intra-molecular and/or solvation motions within the MLCT manifold. It is also possible that the isotropic XSS signal is contaminated by a small amount with the ultrafast OKE signal due to imperfect anisotropic/isotropic decomposition. However, we emphasize that these uncertainties in the early dynamics do not influence the interpretation of the solute dynamics at later times in any way.

Comparison of the 2D XSS signal reconstructed from the three SVD components and the 2D XSS signal reconstructed from the global fitting shows a very good agreement (Supplementary Figure 30). XSS signals derived from the global analysis and related to each time-dependent component are shown in Supplementary Figure 31.

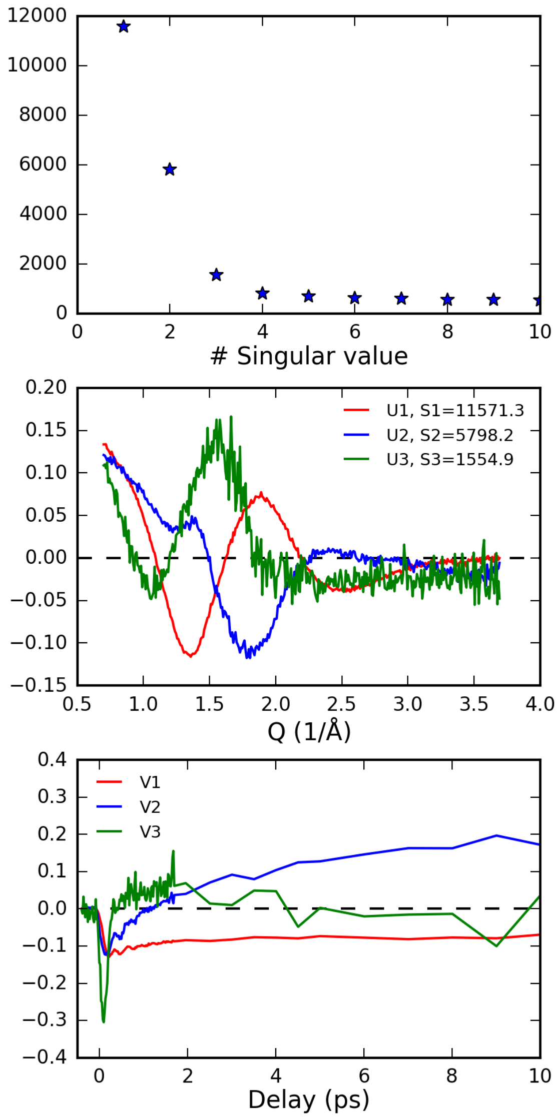

Supplementary Figure 28. Singular value decomposition (SVD) of the $[Fe(bmip)_2]^{2+}$ isotropic XSS data. (Top) Singular values and (middle) the first three respective column vectors U and (bottom) row vectors V.

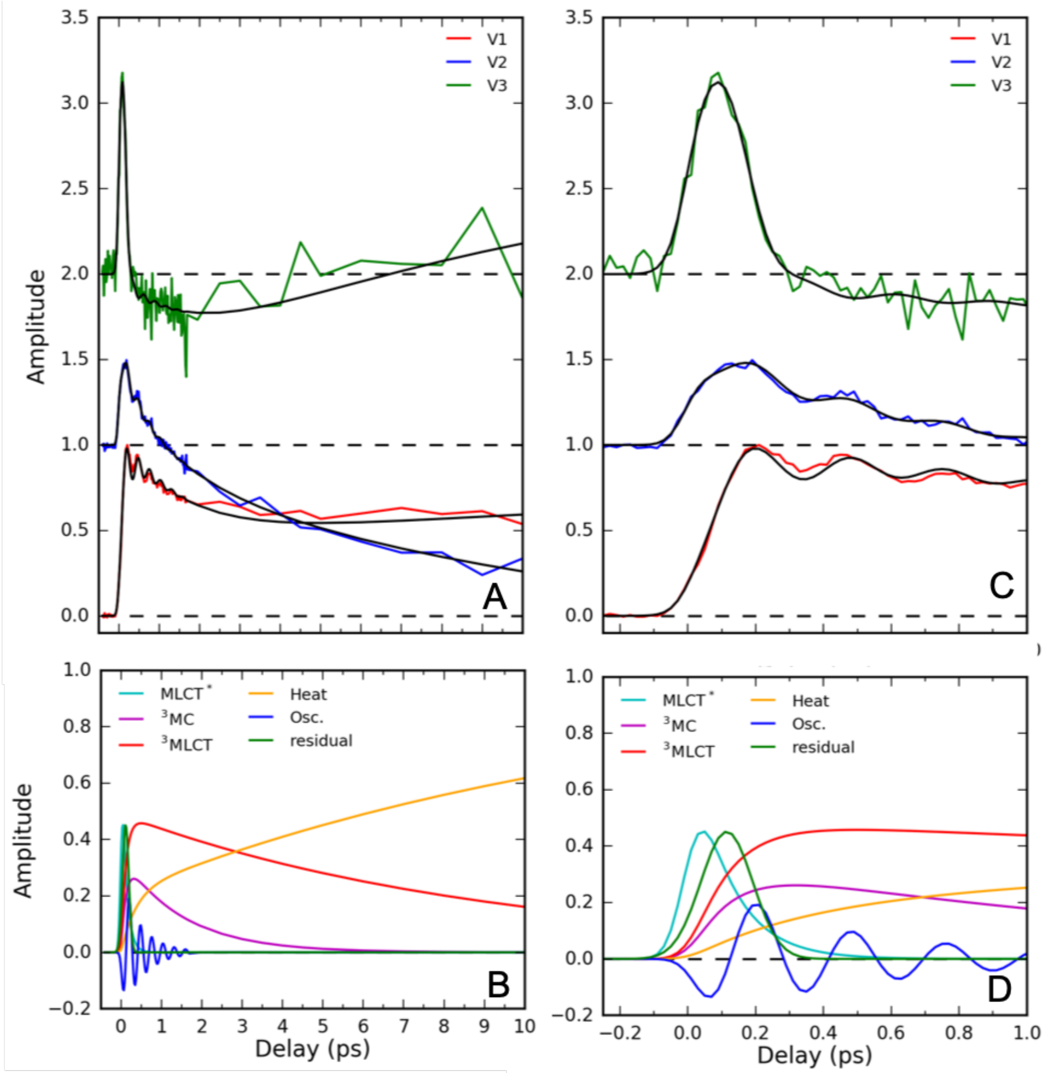

Supplementary Figure 29. Global analysis of the XSS data. (A,C) Fits of the three row vectors V with (B,D) the six time-dependent components.

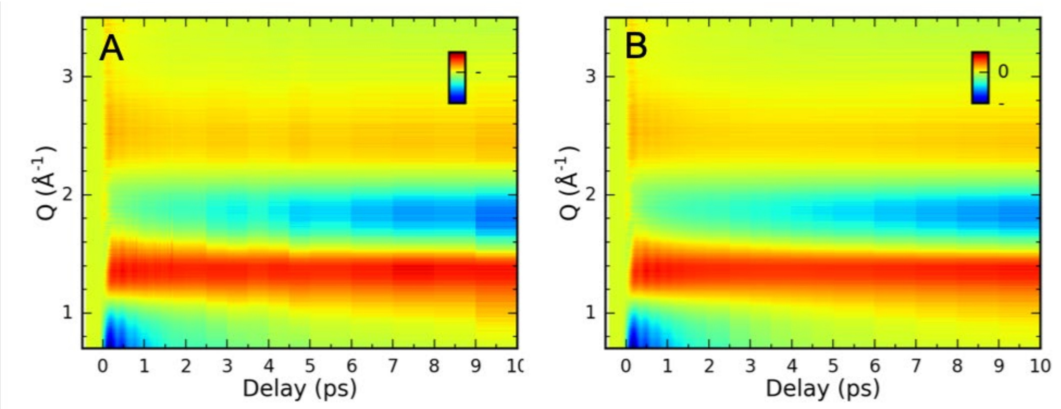

Supplementary Figure 30. Global analysis of the XSS data. (A) XSS signal reconstructed from the three SVD components. (B) XSS signal reconstructed from the six time-dependent components of the global fitting model.

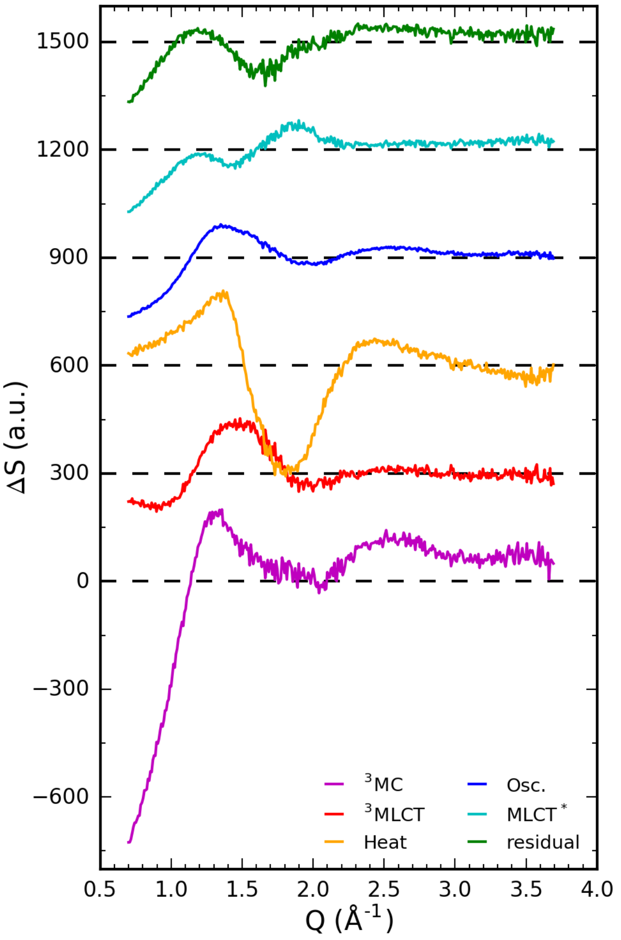

Supplementary Figure 31. XSS signals corresponding to the six time-dependent components derived from the global analysis (Supplementary Figure 29B,D).

Supplementary Note 12: Comparison of different damping mechanisms

Both the solute XSS signal and the Kα XES intensity dependence from the average Fe-ligand bond length *R* is linear. However, because the wavepacket follows a broad distribution of distances *g(R,t)*, then this can influence the observed signals (e.g. broadening of the Kα XES spectrum in addition to the shift). In order to evaluate these effects we calculated the observed signals for three different *g(R,t)*, but with the same ensemble average <*R(t)*> (Supplementary Figure 32). We found no effect to the simulated signals depending on whether the damping mechanism is inelastic (cooling, Supplementary Figure 32A-C) or elastic (Supplementary Figure 32D-F). In the third case where $g(R,t)=\delta(R-\langle R(t)\rangle)$, we found only very small differences from the two previous cases at early time delays <0.3 ps (Supplementary Figure 32G-I). Therefore, the observed signals depend only on the <*R(t)*> and any effects due to the distribution of bond lengths can be omitted.

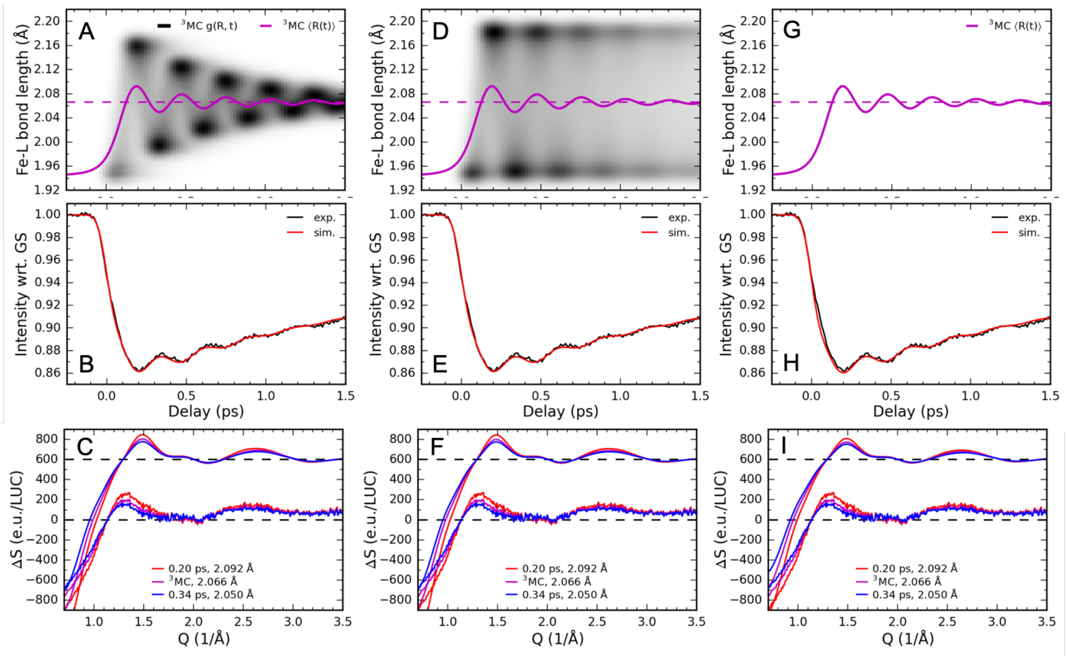

Supplementary Figure 32. Comparison of the different wavepacket dynamics damping mechanisms (A, D, G) to the oscillatory Kα XES (B, E, H) and XSS signals (C, F, I). Left column: simulation with inelastic damping mechanism of *g(R,t)* (vibrational cooling). Middle column: simulation with elastic damping mechanism of *g(R,t)* (dephasing). Right column: simulation with only the ensemble average Fe-L distances <*R(t)*>. In all the simulations oscillation period is 278 fs and the exponential damping time constant is 500 fs. Note that g(R,t) also decays with the 1.5 ps lifetime of the ^{3}MC state.

Supplementary Note 13: Simulations of the XSS signal components

The solute and solvent cage XSS signals of the ^{3}MC state were simulated based on the calculated DFT structures from Ref. [8] (included in Supplementary Note 19). Supplementary Figure 33A shows the dependence of the solute XSS signals from the average Fe-ligand distance if solvent is not included. Supplementary Figure 33A shows the solvent cage signal retrieved from molecular dynamics (MD) simulations for the GS and the ^{3}MC state in their optimal geometries (R = 1.943 Å and R = 2.066 Å, respectively). In the MD simulations, the GS and the ^{3}MC DFT structures of $[Fe(bmip)_2]^{2+}$ were solvated in a cubic box (50 Å size) of acetonitrile molecules using the three-site interaction potential derived by Guardia et al. [9]. The complex is frozen at the optimized geometry by applying harmonic positional restraints with force constant of 1000 kcal/mol and the parameters for intermolecular interactions are taken from the OPLS 2005 force field [10]. MD trajectories were calculated with a Nose-Hoover thermostat at 300 K for total time of 5 ns, with a time step of 2 fs. Frames were saved every ps and subsequently used to calculate the RDFs, which were sampled with a radial bin of 0.01 Å. For these simulations, we use the Desmond software package developed at D. E. Shaw Research [11].

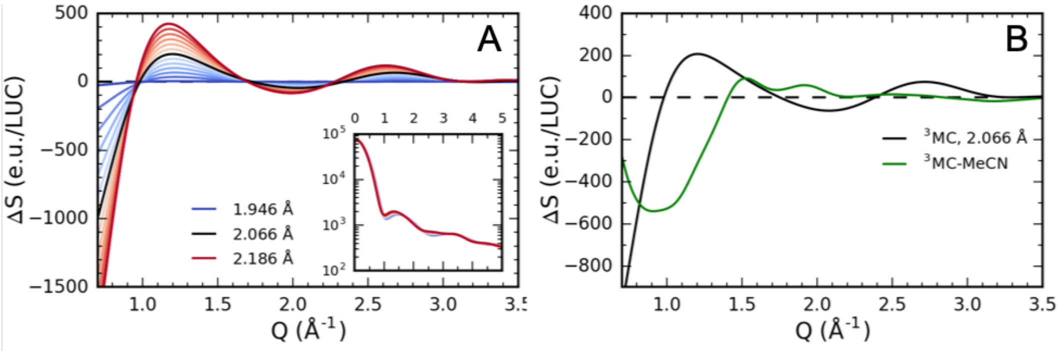

Supplementary Figure 33. Simulated ^{3}MC XSS signals (A) Dependence of $[Fe(bmip)_2]^{2+}$ solute difference XSS signal from the average Fe-ligand bond length (inset without GS subtraction). (B) Calculated solute difference XSS signal for the optimal $[Fe(bmip)_2]^{2+}$ ^{3}MC structure (black) and respective solvent cage difference XSS signal (green).

Supplementary Note 14: RASSCF orbital covalencies

Metal-ligand covalencies of the nominally Fe 3d eg orbital and the nominally ligand occupied σ orbital retrieved from the RASSCF calculations are shown in Supplementary Table 1. Covalencies are calculated for the optimal GS and ^{3}MC geometries and for valence and 1s and 2p core-ionized states. The σ-donation is decreased in the ^{3}MC state with respect to the GS. Also, the covalency is higher in core hole states than in the valence states.

Supplementary Table 1. Percent of Fe 3d character in e_g and σ orbitals for different states and geometries.

Orbital	State	GS geometry	^{3}MC geometry
e_g	^{3}MC, no core hole	77.3	89.9
	^{3}MC, 1s core hole	73.3	79.3
	^{3}MC, 2p core hole	72.3	79.2
σ	^{3}MC, no core hole	18.7	9.2
	^{3}MC, 1s core hole	22.4	15.7
	^{3}MC, 2p core hole	21.8	15.3

Supplementary Note 15: RASSCF core-ionized PES

RASSCF energies of the core-ionized states (nominally with respect to the optimal ^{3}MC state energy), together with the core-ionized PESs retrieved from parabolic fits are shown in Supplementary Figure 34. Force constants of all the PESs is very similar: k_{1s} = 38.0 eV/Å^2 and k_{2p} = 37.9 eV/Å^2 (latter corresponds to the average 2p PES in Supplementary Figure 34, solid gray line). However, the 1s and 2p PESs are displaces with respect to each other. Minimum energy position of the PESs is respectively R_{1s}=2.032 Å and R_{2p}=2.046 Å (latter corresponds again to the average 2p PES). The displacement between the 1s and 2p core-ionized PESs is therefore ΔR_{core}=0.014 Å. Because of this displacement, the energy difference between the states changes with a slope $\kappa_{core}=k_{core}\Delta R_{core}$, where κ_{core} is first-order vibronic coupling and $k_{core}\equiv k_{1s}=k_{2p}$.

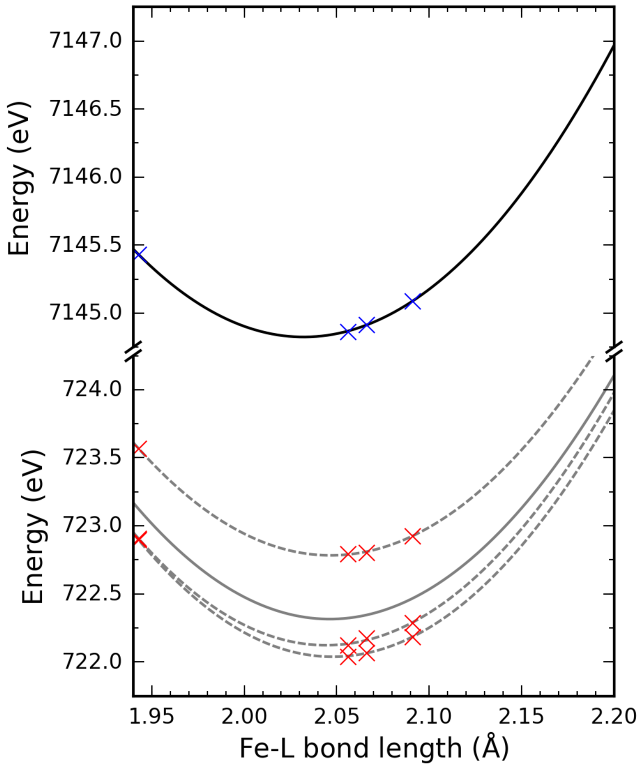

Supplementary Figure 34. Calculated core-ionized state PESs. Blue crosses are the calculated RASSCF energies of the 1s core-ionized states. Solid black line is a parabolic fit to these energies. Red crosses are the calculated RASSCF energies of a three 2p core-ionized states that contribute most to the intensity of the Kα_1 XES spectrum. Dashed gray lines are parabolic fits to these energies. Solid gray line is the average of the three 2p core-ionized PES.

Supplementary Note 16: Equations of time-dependent signals

Functions used to describe the time-dependence of Kα XES, Kβ XES, XSS and UV-vis TA data are presented below. System of kinetic rate equations that define the excited state populations is:

$$\begin{cases} \frac{dN_1}{dt} = -k_1 N_1 \\ \frac{dN_2}{dt} = rk_1 N_1 - k_2 N_2 \\ \frac{dN_3}{dt} = (1-r)k_1 N_1 - k_3 N_3 \\ \frac{dN_4}{dt} = k_2 N_2 + k_3 N_3 - k_4 N_4 \\ \frac{dN_5}{dt} = k_4 N_4 \end{cases} \quad (1)$$

For the clarity of the presentation we have defined here 1 = MLCT*, 2 = ^{3}MC, 3 = ^{3}MLCT and 4 = recovered hot GS, 5 = recovered cold GS. Respective rate constants are $k_{1,2,3,4,5}$. r is the branching ratio between ^{3}MC and ^{3}MLCT. The boundary conditions are:

$$\begin{cases} N_1(t=0) = N_{exc} \\ N_2(t=0) = 0 \\ N_3(t=0) = 0 \\ N_4(t=0) = 0 \\ N_5(t=0) = 0 \end{cases} \quad (2)$$

where N_{exc} corresponds to the initial photoexcited population. Additionally, $N_{1,2,3,4,5}(t < 0) = 0$. Solutions of the above system of equations are convoluted with a Gaussian instrument response function

$$p_{exc}(t) = \frac{1}{c\sqrt{2\pi}} exp\left(-\frac{(t-t_0)^2}{2c^2}\right) \quad (3)$$

Where t_0 is time-zero and $c = FWHM/\left(2\sqrt{2ln2}\right)$ is standard deviation. Thus, excited state populations are

$$P_i(t) = (p_{exc} * N_i)(t) \quad (4)$$

, i=1,2,3,4,5. Photoionized population present during the Kα/Kβ XES and XSS experiment is described by

$$P_6(t) = 0.5 r_{ion} N_{exc}\left(erf\left(\frac{t-t_0}{\sqrt{2}c}\right) + 1\right) \quad (5)$$

Where r_{ion} is the ratio of photoexcited population that is ionized. In this case, other populations are rescaled:

$$P_i(t) \rightarrow (1 - r_{ion})P_i(t) \quad (6)$$

, i=1,2,3,4,5. The GS population that is not photoexcited is

$$P_0(t) = 0.5 N_{exc}\left(1 - erf\left(\frac{t-t_0}{\sqrt{2}c}\right)\right) + 1 - N_{exc} \quad (7)$$

Note that P are defined as relative populations and therefore $\sum P_i(t) = 1$ at any t.
Oscillatory signal is described by a damped harmonic oscillation:

$$A_{osc}(t) = cos(\omega t) exp\left(-\frac{t}{\tau_{damp}}\right) \quad (8)$$

Here $\omega = 2\pi/T$, T is the period and τ_{damp} is the damping time constant of the oscillations. Additionally, $A_{osc}(t < 0) = 0$ and convolution with the MLCT* population is carried out

$$I_{osc}(t) = (p_1 * A_{osc})(t) \quad (9)$$

Here p_1 is the normalized MLCT* population $p_1(t) = P_1(t)/\int P_1(t)dt$.
Solvent heat signal present in XSS is described by two exponential rise times:

$$I_{heat}(t) = \sum_{i=1,2} r_i (p_{exc} * A_i)(t) \quad (10)$$

Where r_i are the relative ratios of the two rise times and

$$A_i(t) = 1 - \exp\left(-\frac{t}{\tau_i}\right) \quad (11)$$

where τ_i is the exponential rise time and $A_i(t < 0) = 0$. The residual ultrafast signal present in the isotropic XSS signal at short time scales is described by a Gaussian function:

$$I_{residual}(t) = A_{residual} exp\left(-\frac{(t-t_{residual})^2}{2c_{residual}^2}\right) \quad (12)$$

Where $A_{residual} = max\{P_1(t)\}$. $t_{residual}$ and $c_{residual}$ are time delay and standard deviation of the ultrafast residual signal.

Time-dependent intensity traces of Kα XES and Kβ XES are modeled as

$$I^{K\alpha,K\beta}(t) = (1 - r_{ion}) \sum_{i=0}^{5} I_i^{K\alpha,K\beta} P_i(t) + I_6^{K\alpha,K\beta} P_6(t) + I_{osc}^{K\alpha,K\beta} I_{osc}(t) \quad (13)$$

$I_i^{K\alpha,K\beta}$ is the intensity associated with the time-dependent component i. Note that $I_0^{K\alpha,K\beta} = I_4^{K\alpha,K\beta} = I_5^{K\alpha,K\beta}$ and $I_3^{K\alpha,K\beta} = I_6^{K\alpha,K\beta}$.

Row vectors V retrieved from the singular-value decomposition of the difference XSS data are model as

$$V^j(t) = (1 - r_{ion}) \sum_{i=1}^{4} V_i^j P_i(t) + V_{osc}^j I_{osc}(t) + V_{heat}^j I_{heat}(t) + V_{OKE}^j I_{OKE}(t) \quad (14)$$

V_i^j is the amplitude associated with the time-dependent component i (different for each row vector V^j, j=1,2,3). Note that $P_6(t)$ is excluded because it very small contribution is effectively included in the solvent heat term $V_{heat}^j I_{heat}(t)$.

Row vectors V retrieved from the singular-value decomposition of the UV-vis TA difference data in Section 6 are model as

$$V^j(t) = \sum_{i=1}^{5} V_i^j P_i(t) \quad (15)$$

Supplementary Note 17: Simulation of the ^{3}MC wavepacket dynamics

Nuclear wavepacket dynamics on the ^{3}MC surface is simulated with a single-mode quantum harmonic oscillator. Dynamics is calculated using the Lindblad master equation

$$\frac{\partial\rho}{\partial t} = -\frac{i}{\hbar}[H,\rho] + \sum_j \left(2C_j\rho C_j^+ - \rho C_j C_j^+ - C_j^+ C_j \rho\right) \quad (16)$$

Here ρ is the density matrix of the system, H is the Hamiltonian of the system and C_j is the collapse operator describing interaction of the system with the environment (j=1,2). Hamiltonian is

$$H = \hbar\omega(n + 0.5) \quad (17)$$

Where $n = aa^+$is number operator and a is annihilation operator. ω is angular frequency of the oscillator. Oscillator period is $T = 2\pi/\omega = 278$ fs ($\hbar\omega \approx 14.9$ meV). Elastic dephasing is described with a following collapse operator

$$C_1 = \sqrt{\gamma_1}n \quad (18)$$

and vibrational cooling is described with

$$C_2 = \sqrt{\gamma_2}a \quad (19)$$

where $\gamma_{1,2}$ are dephasing and cooling rates, respectively. Observed exponential time constants are related to the rates by $\tau = 2\hbar/\gamma$. Initial state is taken to be a coherent Gaussian wavepacket $|\alpha\rangle$, with $\alpha = \sqrt{(m\omega)/(2\hbar)}\,R + i\sqrt{1/(2m\hbar\omega)}\,P$, where R and P are the position and the momentum of the wavepacket and m is the effective mass of the oscillator. In the simulations presented in this manuscript, initial coordinates are R_{init} = 1.943 Å – 2.066 Å = -0.123 Å and P_{init}=0. Value of the effective mass was selected to give the initial energy of the harmonic oscillator $E_{init} = 0.5m\omega^2R_{init}^2 = 0.5\,eV$. Latter corresponds to a reasonable reorganization energy of the ^{3}MC state with respect to the ^{3}MLCT state [12]. For our purpose of calculating the bond length distribution, m is also relevant because the FWHM of coherent Gaussian wavepacket depends on it: $\Gamma = 2.355\sqrt{\hbar/(2m\omega)}$. In the simulation Γ = 0.025 Å, which is a reasonable spread of the initial wavepacket (note however that Γ has no effect to <R(t)>). Lindblad master equation was numerically solved on a harmonic oscillator basis using the QuTiP package (http://qutip.org) [13,14]. Number of vibrational basis functions was 50. This value was selected to be higher than $|\alpha_{init}|^2 + 3|\alpha_{init}|$, where $|\alpha_{init}|^2 \approx 33.7$ and $|\alpha_{init}| \approx 5.8$ are the mean and the standard deviation of the number of vibrational quanta in the initial state, respectively. Evolution of bond length distribution was subsequently calculated with

$$g_0(R,t) = \sum_{i,j}^{50} \rho_{ij}\,(t)\psi_i(R)\psi_j^*(R) \quad (20)$$

where $\psi_i(R)$ are vibrational basis functions (harmonic oscillator eigenstates). In order to take into account population and depopulation of the ^{3}MC state, the $g_0(R,t)$ is appropriately convoluted and weighted with the ^{3}MC decay (defined in the previous Supplementary Note 16):

$$g(R,t) = p_1(t) * [r\exp(-k_2t)g_0(R,t)] \quad (21)$$

The distribution $g(R,t)$ is used to describe the experimentally observed oscillatory wavepacket dynamics (shown in Fig. 5B in the main manuscript and Supplementary Figure 32).

Supplementary Note 18: Formulas describing first-order vibronic coupling

We present below the formulas describing first-order vibronic coupling in a simple one-dimensional case. At a coordinate origin, we define a reference state with a potential energy surface corresponding to a harmonic oscillator:

$$E_{ref}(x) = 0.5kx^2 \tag{22}$$

k is the force constant of the harmonic oscillator and x is the nuclear coordinate. Vibrational frequency of the oscillator is $\omega = \sqrt{k/m}$, m is the effective mass. Let there be a second state with an arbitrary potential energy surface $E(x)$. Without any loss of generality, the energy difference between the states can be expanded into a Taylor series at the coordinate origin:

$$\Delta E(x) \equiv E(x) - E_{ref}(x) = E_0 + \kappa x + \frac{1}{2}\gamma x^2 + \frac{1}{6}\delta x^3 + \dots \tag{23}$$

Here E_0 is the constant energy offset, κ is the first order vibronic coupling, γ is the second order vibronic coupling (change in the force constant) and δ is the third order vibronic coupling (anharmonicity). Below we consider only the first order vibronic coupling κ and therefore the potential energy of the state is:

$$E(x) = E_0 + \kappa x + 0.5kx^2 \tag{24}$$

Note that this formula describes potential energy of a harmonic oscillator under constant external force $F_{ext} = -\kappa$. Energy difference between the states is:

$$\Delta E(x) = E_0 + \kappa x \tag{25}$$

Therefore, energy difference ΔE changes linearly with respect to the nuclear coordinate x and the slope of the change is equal to the first order vibronic coupling κ. Expression of the potential energy E can be rearranged

$$E(x) = E_0' + 0.5k\left(x + \frac{\kappa}{k}\right)^2 \tag{26}$$

Here, the constant energy offset is $E_0' = E_0 - 0.5\kappa^2/k$. Therefore, the first order vibronic coupling stabilizes and displaces the harmonic energy surface. Displacement is equal to

$$x_0 = -\frac{\kappa}{k} \tag{27}$$

This formula is equivalent to Hooke's law. In addition to the displacement, the first order vibronic coupling also determines reorganization energy of the electronic state with respect to the origin:

$$\lambda \equiv E(0) - E(x_0) = 0.5kx_0^2 \tag{28}$$

Reorganization energy is directly related to Stokes shift:

$$\Delta E_{Stokes} \equiv \Delta E(0) - \Delta E(x_0) = 2\lambda \tag{29}$$

Huang-Rhys parameter S is defined as the reorganization energy normalized to the vibrational quantum energy

$$S \equiv \frac{\lambda}{h/(2\pi)\omega} \tag{30}$$

A vertical transition from the reference state vibrational ground state results in a vibronic lineshape given by the Poisson distribution:

$$I(n) = e^{-S}\frac{S^n}{n!} \tag{31}$$

Here n is vibrational quantum number of the final state. The Huang-Rhys parameter S is thus equal to the mean and the variance of the vibronic lineshape and the vibronic broadening can be approximated as

$$\Gamma_{FWHM} \approx 2.355\hbar\omega\sqrt{S} \tag{32}$$

Supplementary Note 19: $[Fe(bmip)_2]^{2+}$ molecular structures

The geometries are from Ref. [8]. The calculations was done at PBE0/6-311G(d,p) level and included acetonitrile solvent through the polarizable continuum model.

Ground state geometry:

63

Fe	0.000001162852	0.000001541627	0.000040245324
C	-0.703153631005	2.975907134636	-2.894741503707
C	-1.927924333090	2.579565326940	-2.496309054496
H	-0.411816152760	3.719789960637	-3.617630317379
C	-1.927354265986	-2.579515035408	2.496882444597
C	-0.702490237543	-2.975887102960	2.894998197515
H	-0.410981733307	-3.719774359325	3.617813869715
C	-3.978425985498	-0.869868828249	0.844185073011
C	-3.978614153500	0.869997967151	-0.843077678218
H	-4.505217283148	-1.547409823400	1.502313318115
H	-4.505547492778	1.547563793673	-1.501068012868
C	0.702437909831	-2.976231312922	-2.894579752291
C	1.927308786323	-2.579847213555	-2.496497097197
H	0.410916418503	-3.720196124495	-3.617310233352
C	1.927975443028	2.579840834907	2.496063739826
C	0.703212159039	2.976251663881	2.894450252538
H	0.411888944167	3.720231969280	3.617244426934
C	3.978632911710	0.870024200066	0.843051114993
C	3.978411506518	-0.870041021397	-0.844006901883
H	4.505578925053	1.547659216170	1.500959980202
H	4.505189826335	-1.547666814715	-1.502058118398
C	2.595058823116	-0.829749038249	-0.804572061499
C	2.595270517549	0.829724232291	0.803982179364
C	-2.595252665627	0.829675476874	-0.804004841784
C	-2.595072344858	-0.829604213625	0.804749721373
C	4.661333456002	-0.000007772396	-0.000567254249
H	5.744746623102	-0.000005580778	-0.000711729594
C	-4.661331187816	0.000078451352	0.000643535209
H	-5.744744358317	0.000100402224	0.000786980786
N	-1.924451491176	0.000022784106	0.000283712920
N	1.924453551425	-0.000015979215	-0.000202811837
C	0.360545994430	-1.366039028541	-1.347358998851
C	0.360902394240	1.366051064229	1.347329964486
C	-0.360874174761	1.365891970267	-1.347422171445
C	-0.360569611787	-1.365884489087	1.347586378809
H	-2.908432372603	2.903814094337	-2.799793674051
H	-2.907790166781	-2.903741748226	2.800624632370
H	2.908489609792	2.904116649920	2.799499757582
H	2.907738917566	-2.904140264318	-2.800187219309
N	-0.231844207566	-2.228269648912	-2.190577951208
N	1.705425855527	-1.593541106344	-1.549384815306
N	1.705836658092	1.593530033203	1.549017351570
N	-0.231261113268	2.228299555307	2.190691181997
N	0.231305534942	2.228025449502	-2.190888765826
N	-1.705804077916	1.593369369461	-1.549138523282
N	-1.705453963700	-1.593326808871	1.549651194213
N	0.231805320789	-2.228032639502	2.190900390396
C	1.667322484656	2.390237579349	-2.337645660939

H	2.089486936159	2.879624653184	-1.458715911444
H	2.138318396273	1.419382911272	-2.482620861872
H	1.861913619440	3.007064795680	-3.213045343151
C	-1.667275075049	2.390541759502	2.337443767930
H	-2.089457814516	2.879745931704	1.458421823918
H	-2.138271054035	1.419718648432	2.482633334791
H	-1.861846634239	3.007554231414	3.212717083107
C	-1.667896671573	-2.390477578684	-2.336985334504
H	-2.089878537855	-2.879709016433	-1.457881836366
H	-2.138905197984	-1.419638162237	-2.482022007276
H	-1.862690728333	-3.007450999100	-3.212236470207
C	1.667854736012	-2.390269884550	2.337303911666
H	2.089798655239	-2.879666148211	1.458273309664
H	2.138900848909	-1.419422471945	2.482163577618
H	1.862645844215	-3.007100436927	3.212656461250

^{3}MC geometry:

63

Fe	-0.09980900	-0.00019000	-0.00004300
C	-0.89793400	2.96012500	-2.92440100
C	-2.11609500	2.54488700	-2.52368400
H	-0.61812600	3.70244100	-3.65340400
C	-2.11566500	-2.54452200	2.52471600
C	-0.89743400	-2.95990800	2.92507600
H	-0.61750600	-3.70217700	3.65408000
C	-4.18307600	-0.86557900	0.86285800
C	-4.18322300	0.86590500	-0.86152700
H	-4.72391700	-1.52949400	1.52338300
H	-4.72417100	1.52980200	-1.52198000
C	0.95784700	-3.00119100	-2.95629400
C	2.15097000	-2.55991100	-2.51340900
H	0.71703000	-3.74963000	-3.69323300
C	2.15141300	2.55945200	2.51320500
C	0.95839200	3.00081300	2.95627600
H	0.71772800	3.74909900	3.69342200
C	4.14207100	0.86748000	0.85032900
C	4.14195500	-0.86677900	-0.85196900
H	4.67750500	1.53830200	1.50866000
H	4.67731000	-1.53714000	-1.51083100
C	2.75620600	-0.81994800	-0.80464200
C	2.75630600	0.81980000	0.80405300
C	-2.79828600	0.82207900	-0.81983600
C	-2.79814800	-0.82183800	0.82084800
C	4.82155700	0.00053400	-0.00104600
H	5.90533300	0.00087400	-0.00146800
C	-4.85444300	0.00020400	0.00076100
H	-5.93838700	0.00024200	0.00088900
N	-2.16195700	0.00014700	0.00047500
N	2.10556200	-0.00031900	0.00000200
C	0.50994600	-1.41792900	-1.40439500
C	0.51013200	1.41776500	1.40423900
C	-0.52708200	1.36808300	-1.36193900
C	-0.52685200	-1.36845300	1.36196200
H	-3.10079400	2.85012400	-2.83388300
H	-3.10030800	-2.84953500	2.83531500

H	3.15229200	2.84659200	2.78641500
H	3.15178100	-2.84736600	-2.78653700
N	-0.01992500	-2.29531400	-2.27064300
N	1.86602200	-1.59157500	-1.56453500
N	1.86625100	1.59125400	1.56425600
N	-0.01952700	2.29508700	2.27067700
N	0.04492200	2.23477200	-2.21054600
N	-1.88310100	1.57153200	-1.56746700
N	-1.88283200	-1.57150400	1.56812100
N	0.04529600	-2.23508000	2.21052900
C	1.47985400	2.41348100	-2.34983500
H	1.89476000	2.85317300	-1.44206800
H	1.95530600	1.45319900	-2.54438900
H	1.66981900	3.08077700	-3.18843200
C	-1.43945100	2.49476700	2.49055900
H	-1.88803314	2.97443580	1.61953963
H	-1.90629523	1.52670178	2.64833493
H	-1.59697929	3.11960238	3.37112306
C	-1.43988400	-2.49411800	-2.49110000
H	-1.88902938	-2.97307643	-1.62000568
H	-1.90606558	-1.52581960	-2.64942899
H	-1.59758347	-3.11925719	-3.37144229
C	1.48025800	-2.41377000	2.34957600
H	1.89527800	-2.85165100	1.44099700
H	1.95556800	-1.45378800	2.54599700
H	1.67026900	-3.08267500	3.18687700

Supplementary References

[1] W. Zhang, R. Alonso-Mori, U. Bergmann, C. Bressler, M. Chollet, A. Galler, et al., Tracking excited-state charge and spin dynamics in iron coordination complexes., Nature. 509 (2014) 345–348. doi:10.1038/nature13252.

[2] Y. Liu, T. Harlang, S.E. Canton, P. Chábera, K. Suárez-Alcántara, A. Fleckhaus, et al., Towards longer-lived metal-to-ligand charge transfer states of iron(ii) complexes: an N-heterocyclic carbene approach, Chem. Commun. 49 (2013) 6412. doi:10.1039/c3cc43833c.

[3] D. Leshchev, T.C.B. Harlang, L.A. Fredin, D. Khakhulin, Y. Liu, E. Biasin, et al., Tracking the picosecond deactivation dynamics of a photoexcited iron carbene complex by time-resolved X-ray scattering, Chem. Sci. 9 (2018) 405–414. doi:10.1039/C7SC02815F.

[4] W. Gawelda, A. Cannizzo, V.-T. Pham, F. van Mourik, C. Bressler, M. Chergui, Ultrafast Nonadiabatic Dynamics of [Fe II (bpy) 3] 2+ in Solution, J. Am. Chem. Soc. 129 (2007) 8199–8206. doi:10.1021/ja070454x.

[5] E. Biasin, T.B. van Driel, G. Levi, M.G. Laursen, A.O. Dohn, A. Moltke, et al., Anisotropy enhanced X-ray scattering from solvated transition metal complexes, J. Synchrotron Radiat. 25 (2018) 306–315. doi:10.1107/S1600577517016964.

[6] D. McMorrow, W.T. Lotshaw, Intermolecular dynamics in acetonitrile probed with femtosecond Fourier-transform Raman spectroscopy, J. Phys. Chem. 95 (1991) 10395–10406. doi:10.1021/j100178a029.

[7] K.S. Kjær, T.B. van Driel, J. Kehres, K. Haldrup, D. Khakhulin, K. Bechgaard, et al., Introducing a standard method for experimental determination of the solvent response in laser pump, X-ray probe time-resolved wide-angle X-ray scattering experiments on systems in solution, Phys. Chem. Chem. Phys. 15 (2013) 15003–15016. doi:10.1039/C3CP50751C.

[8] L.A. Fredin, M. Pápai, E. Rozsályi, G. Vankó, K. Wärnmark, V. Sundström, et al., Exceptional Excited-State Lifetime of an Iron(II)– *N* -Heterocyclic Carbene Complex Explained, J. Phys. Chem. Lett. 5 (2014) 2066–2071. doi:10.1021/jz500829w.

[9] E. Guàrdia, R. Pinzón, J. Casulleras, M. Orozco, F.J. Luque, Comparison of Different Three-site Interaction Potentials for Liquid Acetonitrile, Mol. Simul. 26 (2001) 287–306. doi:10.1080/08927020108024509.

[10] J.L. Banks, H.S. Beard, Y. Cao, A.E. Cho, W. Damm, R. Farid, et al., Integrated Modeling Program, Applied Chemical Theory (IMPACT), J. Comput. Chem. 26 (2005) 1752–1780. doi:10.1002/jcc.20292.

[11] K.J. Bowers, F.D. Sacerdoti, J.K. Salmon, Y. Shan, D.E. Shaw, E. Chow, et al., Molecular dynamics---Scalable algorithms for molecular dynamics simulations on commodity clusters, in: Proc. 2006 ACM/IEEE Conf. Supercomput. - SC '06, ACM Press, New York, New York, USA, 2006: p. 84. doi:10.1145/1188455.1188544.

[12] M. Pápai, G. Vankó, T. Rozgonyi, T.J. Penfold, High-Efficiency Iron Photosensitizer Explained with Quantum Wavepacket Dynamics, J. Phys. Chem. Lett. 7 (2016) 2009–2014. doi:10.1021/acs.jpclett.6b00711.

[13] J.R. Johansson, P.D. Nation, F. Nori, QuTiP: An open-source Python framework for the dynamics of open quantum systems, Comput. Phys. Commun. 183 (2012) 1760–1772. doi:10.1016/J.CPC.2012.02.021.

[14] J.R. Johansson, P.D. Nation, F. Nori, QuTiP 2: A Python framework for the dynamics of open quantum systems, Comput. Phys. Commun. 184 (2013) 1234–1240. doi:10.1016/J.CPC.2012.11.019.
